# The response of ants to climate change

**DOI:** 10.1111/gcb.16140

**Published:** 2022-03-11

**Authors:** Catherine L. Parr, Tom R. Bishop

**Affiliations:** ^1^ Department of Earth, Ocean and Ecological Sciences University of Liverpool Liverpool UK; ^2^ Department of Zoology and Entomology University of Pretoria Pretoria South Africa; ^3^ School of Animal, Plant and Environmental Sciences University of the Witwatersrand Wits South Africa; ^4^ 2112 School of Biosciences Cardiff University Cardiff UK

**Keywords:** CO_2_, eusociality, mutualisms, physiology, plasticity, precipitation, temperature, thermal tolerance

## Abstract

Ants (Hymenoptera: Formicidae) are one of the most dominant terrestrial organisms worldwide. They are hugely abundant, both in terms of sheer numbers and biomass, on every continent except Antarctica and are deeply embedded within a diversity of ecological networks and processes. Ants are also eusocial and colonial organisms—their lifecycle is built on the labor of sterile worker ants who support a small number of reproductive individuals. Given the climatic changes that our planet faces, we need to understand how various important taxonomic groups will respond; this includes the ants. In this review, we synthesize the available literature to tackle this question. The answer is complicated. The ant literature has focused on temperature, and we broadly understand the ways in which thermal changes may affect ant colonies, populations, and communities. In general, we expect that species living in the Tropics, and in thermally variable microhabitats, such as the canopy and leaf litter environments, will be negatively impacted by rising temperatures. Species living in the temperate zones and those able to thermally buffer their nests in the soil or behaviorally avoid higher temperatures, however, are likely to be unaffected or may even benefit from a changed climate. How ants will respond to changes to other abiotic drivers associated with climate change is largely unknown, as is the detail on how altered ant populations and communities will ramify through their wider ecological networks. We discuss how eusociality may allow ants to adapt to, or tolerate, climate change in ways that solitary organisms cannot and we identify key geographic and phylogenetic hotspots of climate vulnerability and resistance. We finish by emphasizing the key research questions that we need to address moving forward so that we may fully appreciate how this critical insect group will respond to the ongoing climate crisis.

## Graphical abstract

In this review, we synthesise available literature on how climate change drivers will affect ants from the individual to the community. We highlight that eusociality may confer greater adaptation and tolerance to climate change than for solitary organisms. We identify key geographic and phylogenetic hotspots of climate change risk and resistance.

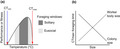

## 1 INTRODUCTION

Ants are a globally important insect group. They evolved in the Cretaceous up to 168 million years ago (Moreau et al., 2006) and have come to occupy nearly all terrestrial microhabitats and trophic positions (Hölldobler & Wilson, 1990; Parker & Kronauer, 2021). They currently number more than 17,000 described ant species (AntWeb, www.antweb.org), are found on all continents except Antarctica, and dominate terrestrial ecosystems in terms of their abundance and biomass (Tuma et al., 2020). Ant biology is underpinned by a eusocial lifestyle—they live in colonies where sterile workers gather resources to provision the reproductive caste. While many ant colonies live in a single, static nest with a sole reproductive queen, a myriad of variations on this basic eusocial plan have evolved leading to nomadic, parasitic, agricultural, and aggressively territorial life‐history strategies (Hölldobler & Wilson, 2009).

Across the globe, ants are considered key contributors to many ecosystem processes, including seed dispersal, pest control, and soil bioturbation, and are thought to structure invertebrate communities via predation and competition (Del Toro et al., 2012). In consequence, changes to ant abundance and occurrence patterns due to ongoing climate change are likely to have significant implications for the structure, integrity, and functioning of terrestrial ecosystems.

**FIGURE 1:**
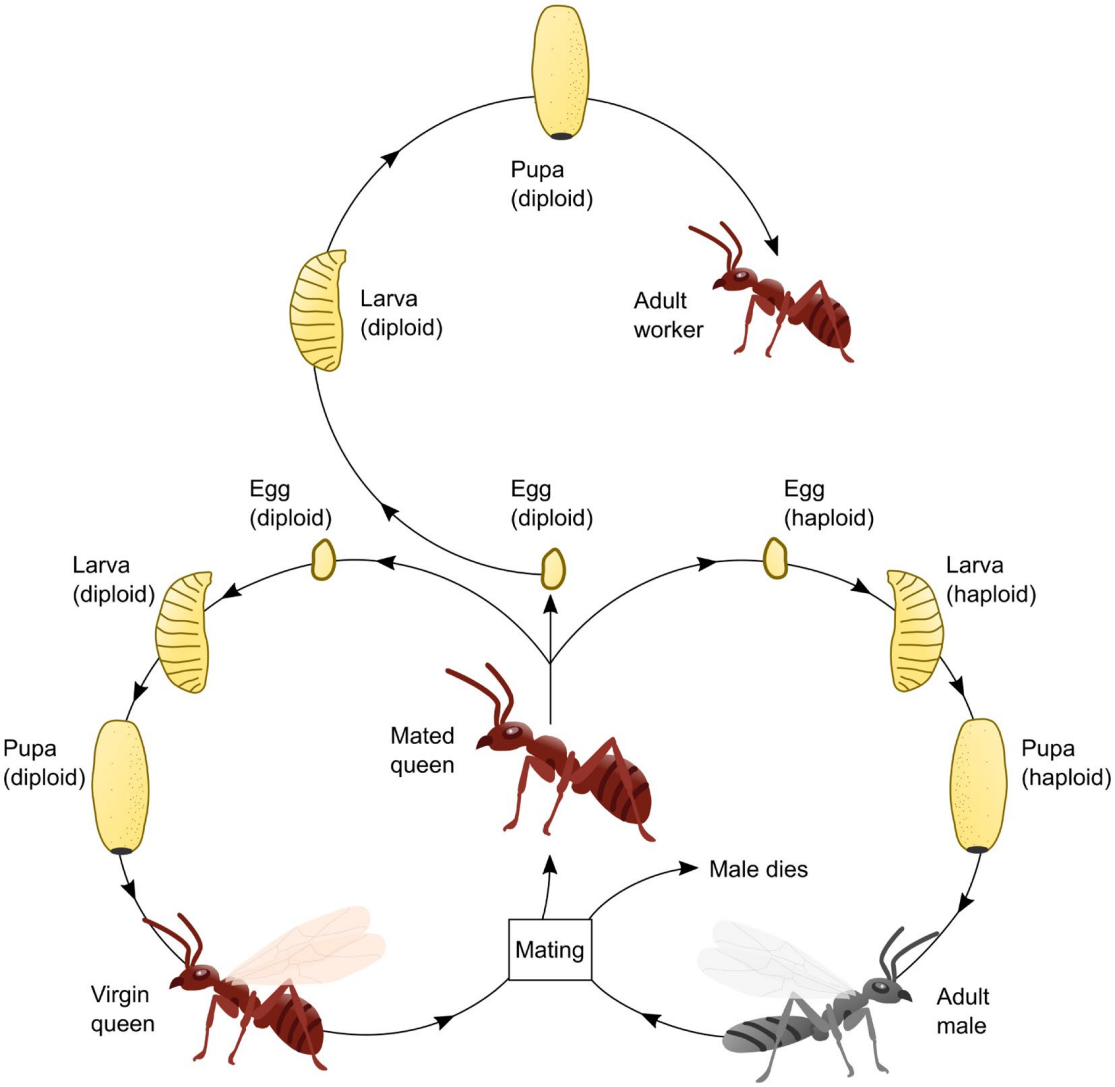
Typical ant life cycle. Mated queens shed their wings, found a new colony, and lay either diploid (fertilised) or haploid (unfertilised) eggs. Haploid eggs develop into males, who die after mating. Diploid eggs develop into workers or new queens. The development cue which triggers queen development varies by species but can be nutritional, pheromonal, or environmental. All ants are holometabolous and undergo full metamorphosis from egg, to larva, to pupa, to adult ant. Based on figure 5.9 from Hölldobler and Wilson (2009)

In this review, we summarize the state of knowledge on how ant colonies, populations, species, and communities will respond to climate change. Crucially, we argue that the eusocial nature of ants will greatly influence their response to present and future climate change. Although ants are holometabolous insects—undergoing full metamorphosis (Figure 1)—their sociality provides them with a behavioral and phenotypic plasticity that may allow many species to escape the effects of climate change in ways that solitary species cannot. This is because ants have both their individual, holometabolous lifecycle, and their social, colony lifecycle. As a result, the climatic challenges that social individuals face represent different balances of risk and reward compared with solitary organisms. We begin by reviewing the literature on the direct and indirect climate change effects that ants are likely to face and outline the geographic and phylogenetic contexts where ants may be particularly vulnerable or resistant to climate change (Sections 2, 4). We continue by considering how the social nature of ants will likely have a major, yet largely unappreciated, influence on their response to climate change (Sections 5, 6). We then explore the wider ecosystem consequences of predicted changes (Section 7), before highlighting the major research questions that should be tackled moving forward (Section 8).

We focus on native ants in this review. This is because there have been recent reviews on climate change and invasive ants (Bertelsmeier et al., 2015, 2016), and invasive range changes are often driven more by human actions (i.e., jump dispersal), with climatic niche shifts being much less pronounced in invasive than non‐invasive species (Bates et al., 2020).

## 2 DIRECT EFFECTS

The direct effects of climate change include elevated temperature, altered precipitation patterns and increased frequency and severity of extreme weather events (e.g. cyclones), but other abiotic changes include elevated atmospheric CO_2_ and UV. Here for each driver, we describe how these environmental changes will directly impact ants across scales (individuals to populations to communities) and axes (spatial, temporal, and self, Bellard et al., 2012).

### 2.1 Temperature

**FIGURE 2:**
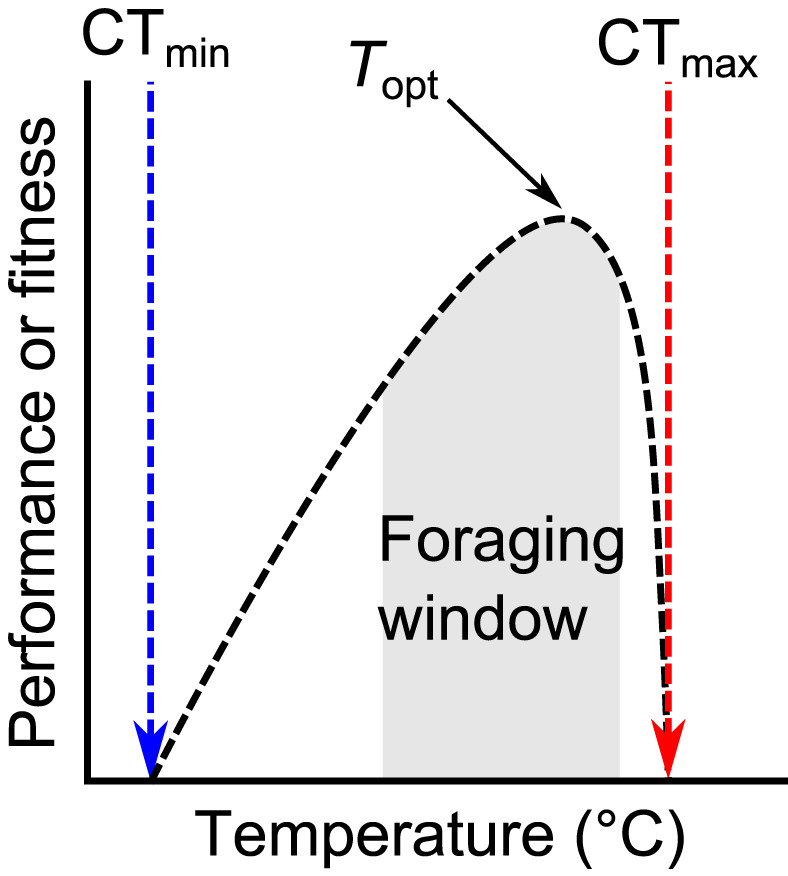
Illustration of a typical thermal performance curve. Critical temperatures (CTmin and CTmax) are illustrated by dashed vertical lines. The perfomance curve is marked as the black dashed curve. Topt indicates the optimum temperature where performance (measured as growth, reproductive success, or some ecological proxy such as running speed) is maximised. The grey shaded area indicates the temperatures over which a hypothetical species may actively forage

Ectotherms are constrained by temperature with ants considered especially sensitive. Ants have been described as thermophilic—or cryophobic (Bishop et al., 2017)—for as long as they have been formally studied (Archibald et al., 2011; Hölldobler & Wilson, 1990). Temperature clearly influences nearly every aspect of ant biology, but the blanket characterization of them as thermophilic is misleading. Ants display a thermal performance curve that is similar to many other poikilothermic ectotherms (Figure 2): ecological performance or fitness increases with temperature up to an maximum level before rapidly declining as temperature becomes too high to allow normal cellular and organismal functioning (Angilletta, 2009). Consequently, at extreme low and high temperatures ant metabolism, performance, and diversity drop to near zero (Hurlbert et al., 2008; Jenkins et al., 2011). Different ant species have adapted the general shape of this performance curve to specialize on a range of extreme temperatures, both hot (Wehner & Wehner, 2011) and cold (Berman et al., 2010). Many researchers have focused on the hot end of this spectrum and describe ants as thermophilic—but no formal comparative analyses between paired ant and non‐ant taxa exist to definitively demonstrate this. We argue that, like many other ectotherms, a better description of ants is that they are a highly *thermally responsive* taxon. Across the globe and their phylogeny, ant individuals, populations, and communities respond quickly and strongly to thermal gradients. This responsiveness has clear implications for the response of ants to ongoing climate change.

At the individual colony level, ants show a high degree of thermal preference in their nesting and brood‐rearing behaviors. For instance, ants frequently choose to nest in species‐specific thermally optimal microsites. They do this by constructing their nests at different depths in the soil column (Bollazzi et al., 2008; Tschinkel, 1987), under differently sized stones (Dean & Turner, 1991), or in exposed areas on bare ground (Pedersen & Boomsma, 1999; Pontin, 1960). Within a given location, these optima are often toward the higher end of available environmental temperatures (Brian & Brian, 1951; Sanada‐Morimura et al., 2006), although not always (Warren et al., 2019). Furthermore, experimental and seasonal changes in temperature can cause ants to relocate their established nests to more suitable thermal environments (Carlson & Gentry, 1973; Heller & Gordon, 2006; McGlynn et al., 2010). While not all nesting behaviors are driven primarily by temperature (Banschbach et al., 1997; McGlynn, 2012), the ability of many ants to choose and move nest sites offers them great flexibility under a range of thermal conditions (Kadochová & Frouz, 2013).

The underlying reason for the ants’ thermal preference in nest site selection is that temperature strongly constrains their metabolism (Shik et al., 2019), development (Porter, 1988), and performance (Hurlbert et al., 2008)—in line with the thermal performance curve (Angilletta, 2009). To boost developmental rates and to complement their broad‐scale nest site selection, ants also take advantage of the hyper‐local thermal gradients that exist within their nests. These local gradients are caused by soil depth and nest aspect. Many species track these intra‐nest thermal gradients and transport their brood from the cooler subterranean chambers to the warmer chambers closer to the soil surface at different times of the day. This allows them to optimize the developmental rate of their brood (Penick & Tschinkel, 2008; Roces & Núñez, 1989; Vanderplank, 1960). This careful manipulation of brood development also extends to plastically altering nest architecture to seasonally increase the surface area directed at the sun (Vogt et al., 2004). These two mechanisms, nest site selection and intra‐nest brood transport, lead to a simple prediction in the light of rising temperatures: many ant species will relocate their nests and alter their brood movement cycles to plastically adapt to warmer thermal regimes. Consequently, through the regulation of nest temperature ants will be able to reduce the negative effects of rising temperatures and may even benefit from them.

Ant thermal sensitivity can also be found outside of the nest and in the daily and seasonal rhythms of their foraging schedules (Hölldobler & Wilson, 1990). For most ant species, foraging above ground is the main mechanism of resource capture. These foraging trips cost resources, however, in terms of energy and worker condition because these environments are thermally exposed and variable. Consequently, ant colonies must avoid the inefficiencies of foraging at low temperatures (where low walking speeds may inhibit effective resource capture), and the lethal risk of foraging at extremely high temperatures. Ants largely select intermediate to high thermal ranges within which to forage, although this differs depending on the local thermal regime. For example, in general, ant foraging activity (i.e., number of foraging workers) is unimodal in many cool environments or during the winter, and peaks at the warmest times of the day (Cros et al., 1997). In hot environments and seasons, a bimodal daily foraging schedule is followed whereby the extreme heat of midday, and the relative cool of dawn and dusk, are avoided (Greenaway, 1981). Consequently, as temperatures rise it likely that many ant species will be able to flexibly adjust their foraging schedules to compensate (Bernstein, 1979; Villalta et al., 2020; Vogt et al., 2004). Indeed, simulation models and empirical data indicate that this alteration of foraging schedules will occur as temperatures rise: cold‐active species may gain more time to forage while warm‐active ones will lose available foraging time (McMunn & Pepi, 2022; Roeder et al., 2022). This flexibility, however, may be constrained in some contexts. For instance, a major narrative in ant ecology is that subordinate species forage at suboptimal temperatures to avoid direct competition with dominant species (Cerdá et al., 1998). Whether these patterns reflect the “ghost of competition past” or current competitive hierarchies, and how common this is remains to be fully resolved (Stuble et al., 2017). If these foraging patterns represent ancient competitive interactions, then “subordinate” species which are now adapted to higher temperatures may benefit from longer periods of optimal foraging time as temperatures rise.

Temperature also shapes the structure of entire ant communities in the wild. In general, warmer locations and time periods host greater ant abundance and species richness. These patterns can be seen at the local scale (Joseph et al., 2019), across elevational gradients (Bishop et al., 2014; Sanders et al., 2007), latitudinal gradients (Dunn et al., 2009; Economo et al., 2018; Gibb et al., 2015; Jenkins et al., 2011), and seasons (Andersen, 1983; Bishop et al., 2014). This link between temperature and community‐level richness at a range of scales and contexts suggests that communities will support a greater diversity of ant species as temperatures rise. The logic here is that environments that were previously inaccessible to many ant species, or were unable to support many colonies, due to their low temperatures will become warmer, opening up immigration and establishment opportunities (Bishop et al., 2019). There is evidence for this idea from both observational and experimental studies. For instance, Kaspari et al. (2019) used observational data from a diversity of natural habitats resampled over 20 years to show that moderate temperature increases (+1°C) increased the number of colonies and species supported at a site. This effect was non‐linear, however, as even greater temperature increases (+2.4°C) reduced colony abundance and species richness (Kaspari et al., 2019). With large temperature increases, especially from a high starting point, it is hypothesized that many populations “tipped over” their thermal optima and into the negative part of their thermal performance curve (Figure 2). This non‐linear impact of temperature on ant communities can also be seen in their geographic distributions. Using species distribution modelling techniques to forecast the future, Fitzpatrick et al. (2011) show how ant diversity will likely increase in temperate regions as temperatures rise but that it will decrease in the Tropics as temperatures become intolerable. This is the thermal performance curve writ large at the community and macrogeographic scale. In the field, increased temperatures have caused upslope shifts in entire ant communities as species climb to higher elevations in an attempt to keep track of their thermal optima (Menke et al., 2014). This highlights how the thermal responsiveness of ants can play out across relatively short (20 year) timescales. Similarly, experimental warming treatments in the eastern USA have shown that higher temperatures can increase colony occupancy rates, decrease colony abandonment and extinction rates, and increase forager abundance and richness (Diamond et al., 2016; Stuble et al., 2013). However, these artificial temperature rises also caused decreases in overall community stability (Diamond et al., 2016). As temperatures rise, we expect that ant communities will largely track their thermal envelopes, moving to higher latitudes and elevations in a similar way to other organisms (Pecl et al., 2017).

Several studies have experimentally manipulated the temperatures that ant communities experience to understand how these new conditions will influence community dynamics and diversity (Diamond et al., 2016; Menke et al., 2014; Pelini et al., 2011). These studies reveal a mixed set of effects: ant communities become less stable following warming (Diamond et al., 2016); community composition changes in orthogonal ways relative to change recorded along natural temperature gradients (Menke et al., 2014); warming may show no effect on diversity and community composition relative to controls (Menke et al., 2014); and diversity in warmed plots can be reduced in sites which are already relatively warm, with no change recorded following warming at relatively cool sites (Pelini et al., 2014). Furthermore, compositional changes following artificial warming, when they occur, can be due to changes in the abundance of less than 10% of the species present (Pelini et al., 2014). The effects of artificial warming appear therefore dependent of species and geographic contexts. These experiments typically warm an area of between 5–10 m in diameter (Menke et al., 2014; Pelini et al., 2011). The experimental logic is that because most of the ant species in these warming chambers do not forage much further than 1 m from their nest, that the warming treatment is manipulating the resident ant communities. This is true in the sense that any ant colonies living in these heat treatments are experiencing elevated temperatures with knock‐on consequences for their foraging ability, growth, and survival. These treatments, however, do not alter the wider population dynamics within which these experimental communities are embedded. Consequently, heat treatments on this scale may simply be acting as sink populations that are constantly fed by immigrants from outside the experiment. Findings from these kinds of studies should therefore be interpreted with caution and should represent a conservative estimate of the effect of rising temperatures on natural ant communities.

Studies on the thermal response of ant communities emphasize that outcomes will be trait dependent. The thermal performance curves of species are different, yet all species in a locality will experience similar temperature changes. Therefore, some species will move up their performance curve closer to their optima, while others will exceed this threshold and experience negative performance and fitness consequences (Figure 2). In this way, different thermal responses can lead to uneven and sometimes idiosyncratic responses at the community level. For instance, biophysics dictates that key features of gross ant morphology, specifically body size and color, have key roles to play in heat gain and loss. In general, larger bodies gain and lose heat more slowly than smaller bodies, and darker colored organisms heat up more rapidly than paler colored ones (Willmer & Unwin, 1981). These relationships appear to play out at the community level: ant species with darker (Bishop et al., 2016) and larger (Bishop et al., 2016; Gibb et al., 2017) workers are more abundant in colder environments across the globe. Consequently, as the climate warms, we may expect to see species with smaller and paler workers coming to dominate ant communities as their phenotypes are more suited to existing in warm conditions. This may lead to the generation of non‐analogous communities in the future as species respond to temperature in different ways dependent on their traits (Bishop et al., 2019). The extent to which these trait‐environment relationships will predict the dynamics of individual species in reality, however, is unclear. Data from birds suggest that there is a disconnect between the trait‐environment relationships of species vs, those of entire communities (Delhey, 2017); therefore, although traits may drive the overall shape of communities, the responses of individual species to their environment are much less predictable.

Similar community‐level patterns can be seen from the perspective of ant thermal physiology. Different ant species have different thermal tolerances. These tolerances are typically estimated as the highest (CT_max_) or lowest (CT_min_) temperature at which individual ants can retain muscular coordination (Roeder et al., 2021b). A general observation is that ants have lower CT_min_ in colder environments and higher CT_max_ in hotter environments (Baudier et al., 2015; Bishop et al., 2017; Diamond et al., 2012; Kaspari et al., 2015; Roeder et al., 2021b). There is potentially large variation, however, in thermal tolerance at the local scale (Kaspari et al., 2015). As environments warm, this variation in thermal tolerance can dictate which species are able to thrive. For instance, (Roeder et al., 2021a) showed that over a 20‐year time period where temperatures rose on average 1°C, ant species with higher CT_max_ values increased their occupancy of local plots over those species with lower CT_max_ values. This effect is probably caused by thermally tolerant species being able to continue foraging under stressful temperatures. Indeed, Stuble et al. (2013) showed that species with higher CT_max_ values were able to maintain higher forager densities under higher temperatures, adding support to this idea. Further, it has been shown that some species with high CT_max_ may also require higher developmental temperatures (Penick et al., 2017). If this effect is general, then rising temperatures may boost the populations of thermally tolerant species by providing more optimal foraging and developmental conditions. The ant thermal physiology literature has been recently reviewed in detail by Roeder et al. (2021b). Consequently, the response of individual ant species to altered temperature regimes will be dependent on their morphological and physiological traits – for instance, there are conflicting reports as to whether larger body sizes also confer more extreme thermal tolerances (Kaspari et al., 2015; Roeder et al., 2021b). Finally, it is unclear how these thermal physiological responses will interact with other physiological tolerances with which they may be co‐adapted (Sinclair et al., 2013). In ants, different trade‐offs between thermal tolerance and desiccation resistance exist in different environments (Bujan et al., 2016), yet few data exist to draw solid conclusions as to how these physiological interdependencies will alter species and community level response.

### 2.2 Carbon dioxide

Rising atmospheric CO_2_ is a pervasive component of climate change. Levels have risen to 416 ppm at the time of writing (August 2021) and are higher than at any point in the past 800,000 years, with the previous highest concentration >300,000 years ago (at 300 ppm) (gml.noaa.gov). Rising atmospheric CO_2_ can have profound effects on plant growth (e.g., C3 plants being particularly responsive), physiology and chemistry (e.g., higher C:N). In contrast, effects on animals have received much less attention and, for ants, the effects are virtually unknown. Recent work by Tocco et al. (2021), however, demonstrated that elevated CO_2_ can increase dung beetle developmental time, increase mortality and result in reduced adult body size and mass. Because many ants also spend a large proportion of their life, including their developmental phase, underground, it is possible they will respond similarly.

Yet, currently CO_2_ levels inside ant nests belowground can be much higher than background levels (e.g., 1–3% in leafcutter nests) and this does not pose a problem (Römer et al., 2017, 2018)—as is the case for some other insects too (e.g., termites, tiger beetles, Tocco et al., 2021). Ants also manage CO_2_ levels via nest architecture and ventilation systems, and ground‐nesting ants can move brood to shallower depths (and therefore lower CO_2_ levels) when necessary (Kleineidam & Roces, 2000; Römer et al., 2018). The ability of ground‐nesting ants to cope with higher CO_2_ has, however, evolved over hundreds of thousands of years. How ants in smaller colonies generally not exposed to as high levels of CO_2_ in the nest (as fewer individuals are respiring) will respond to rapidly rising atmospheric CO_2_ levels remains unknown. Given the consequences have the potential to be quite profound in terms of developmental time increases, this requires greater attention.

### 2.3 Precipitation

Globally temperature is a key determinant of ant diversity, but precipitation also influences ant richness and it can strongly interact with temperature (Jenkins et al., 2011; Szewczyk & McCain, 2016). Although warming is not uniform across the globe, the upward trend in the globally averaged temperature indicates that more areas are warming than cooling. The effects of altered precipitation linked to climate change, however, are much more spatially variable with some areas predicted to experience increased precipitation while other regions will become much drier. Research to‐date has focused on the effects of drought, which are anticipated to increase in frequency and severity in many regions, rather than increased rainfall. These studies suggest in both wet tropical environments and deserts, drought may reduce ant richness and alter species composition (Almeida et al., 2020; Gibb et al., 2019), in part because rainfall moderates ecosystem productivity. Changes to precipitation, in tandem with temperature, are likely to alter humidity and therefore the desiccation risk for ants. In drying environments, ants that are better able to reduce desiccation should be favored. Studies have found that arboreal ants are better adapted to drier conditions in the canopy showing higher desiccation resistance (ability to tolerate water loss) than ground ants (Bujan et al., 2016; Hood & Tschinkel, 1990).

### 2.4 Ultraviolet‐B

The effects of ultraviolet‐B (UVB) are likely to be less significant for the ants than the other changes associated with climate change. The amount of UV radiation received at the Earth's surface is anticipated to change markedly as a consequence of altered cloud, snow and ice cover, and greenhouse gas–induced transport of ozone (Williamson et al., 2014). By 2100, it is predicted that increased cloud at high latitudes (especially in the north) will reduce UV, while the Tropics are predicted to experience an increase in UV because cloud and ozone levels will decline (Williamson et al., 2014). The principal effects of altered UVB are likely to be via morphological changes. As ectotherms, the effects of UVB interact with temperature. With low‐moderate UVB, there is a benefit to being darker in cool locations and paler in hot locations, however, in hot locations with very high UVB, ants tend to have darker pigmentation as protection (Bishop et al., 2016; Law et al., 2020). We might, therefore, expect that where UVB is especially high, assemblage composition will change as darker colored ant species will do better. Canopy ants in the Tropics already have darker pigmentation (Law et al., 2020), so it is unclear how much darker ants can get and whether they will be negatively affected by increased UVB or will simply move into more sheltered microhabitats. At an individual level, ants may develop darker body color, although current available evidence suggests intraspecific variation in ant morphology is not correlated with range size or environment (Gaudard et al., 2019; but see Warren et al., 2016), thus whether ants can adapt to increased UVB remains to be seen.

## 3 INDIRECT AND INTERACTIVE EFFECTS

Generally, many ants appear to be flexible and can adapt behaviors to mitigate the effect of climate change (e.g., movement of brood within nest). However, because ants engage in a multitude of diverse mutualistic interactions varying in strength and importance, there may be important climatic impacts that spread across ecological networks. Perhaps unsurprisingly given the diversity and huge number of ant mutualistic interactions, there has been relatively little work on the potential effects of climate change on these important interactions; this lack of attention matters, however, given how critical many of these mutualisms are for structuring communities and ecosystems.

Broadly speaking most mutualisms relate either to dispersal or defence. Myrmecochory, or seed dispersal by ants, is widespread among plants with more than 3,000 plant species known to have their seeds dispersed in this way (Beattie & Hughes, 2002; Rico‐Gray & Oliveira, 2007). Ants consume the fatty, nutrient‐rich eliaosome, and in return move the seed, often burying it. Because ant foraging is strongly influenced by temperature, particularly for species with lower CT_max_ (e.g., the keystone seed‐dispersing ant, *Aphaenogaster rudis*, in North America), it might be predicted that if foraging windows (Figure 2) are reduced by rising temperatures, the number of seeds dispersed and the distance the seeds are taken will decline. There is, however, little evidence currently to support this. Indeed, in a warming experiment, Stuble et al. (2014) found seed removal rates did not change with experimental warming treatment and they concluded this process may be relatively resistant to climate change.

There is, nevertheless, some suggestion that rainfall may influence myrmecochory. Seed dispersal rates and distances have been found to decrease with increasing aridity (Oliveira et al., 2019); high‐quality seed dispersers appear disproportionately affected which could be problematic where there is low functional redundancy. Conversely, in regions where rainfall is predicted to increase, seed dispersal services may in fact be enhanced, although the extent to which these effects are dependent on relative and absolute rainfall change is unknown.

As with many mutualisms, but particularly for obligate ones, phenological timing is critical and misalignment is highly problematic. For example, seed dispersal outcomes for early vs late flowering plant species may differ because it depends on whether cold or warm‐adapted ant species are foraging. In a novel transplant experiment, Warren and Bradford (2014) found that early blooming plant species were more at risk from asynchronous phenological timing because they were dependent on cold‐adapted species alone whereas later blooming species had their seeds dispersed by both warm‐ and cold‐adapted ants species which enhanced effective seed dispersal.

Ant–plant interactions related to defence are abundant and diverse. Via these facultative and obligate defensive mutualisms, ants protect the plant from herbivory, and, in return, the plants provide rewards such as nutrition (via extrafloral nectaries—EFNs—and Beltian bodies) and nest sites (domatia) (Ness et al., 2010). Climate change research in this field is rare, particularly in relation to temperature effects, although generally it is thought that stress conditions may mean plants invest more in ant defences (Coley et al., 1985) and climate change may enhance the sensitivity of plants with EFNs to anthropogenic disturbance (Arnan et al., 2022). With elevated temperatures in East Africa, the activity of ants defending *Acacia* plants increased, increasing the degree of protection the plant received (Tamashiro et al., 2019); therefore, greater protection from herbivory may result under climate change. Elsewhere this relationship might not be as evident because higher temperatures may shrink the window available for foraging, ultimately decreasing ant activity (e.g., in tropical canopies), reducing ant access to EFNs with consequences for the plant via increased herbivory. This remains to be investigated.

There is some suggestion that overall nectar production from EFNs may not alter with reduced rainfall or elevated CO_2_, but ant protection services may nevertheless be compromised because the composition of attendant ant species can change (Oliveira et al., 2019). Elevated CO_2_ may have a somewhat more complex effect as although the proportion of leaves producing nectar increases, less nectar is produced per active leaf (Fabian et al., 2018)—it is unclear whether this may have the effect of increasing ant defence by increasing the area ants cover, or decreasing defence because rewards are reduced.

Perhaps one of the most well‐known mutualistic interactions ants participate in is with Hempiterans: ants protect aphids and scale insects from predation, while in return are provided with honeydew, a sugar‐rich secretion. These interactions have attracted relatively more attention in relation to climate change, presumably because there are implications for agricultural systems. With elevated temperature and CO_2_, and reduced precipitation, honeydew production can increase, as a result, the frequency with which ants tend aphids increases, ant aggression and colony size can increase and herbivory is reduced (Kremer et al., 2018; Pringle et al., 2013; Zhou et al., 2017)—although this needs weighing against the downside of increased damage by aphids. These effects are most likely to be pronounced in temperate regions where temperatures are not too high (<26°C, Blanchard et al., 2019). Elsewhere, elevated temperatures can cause the breakdown of ant‐aphid mutualisms because ant aggression declines, predators increase and aphid abundance declines (Barton & Ives, 2014), ultimately causing the decline of an agricultural pest. As with seed dispersal, phenological mismatches between hempiterans and ants could disrupt the benefits exchanged (Mooney et al., 2019) and a switch from a mutualistic to antagonistic relationship.

Other nutritional benefits from mutualisms include the less visible. Many species of ants, particularly those from the Camponotini taxonomic group, have bacterial endosymbionts that live within specialized host cells and provide nutritional functions. Ants exposed to high temperatures for 4 weeks had almost complete elimination of their bacterial endosymbiont (*Blochmannia*) (Fan & Wernegreen, 2013)—what remains unknown is how this will play out in natural environments, what the outcome of such endosymbiont depletion would be and whether symbionts may be able to adapt to changing temperatures.

The effects of climate change on mutualistic interactions are among the most complex, challenging and likely the most significant responses to understand, and yet we know so little about them. Clearly, species involved in specific obligate relationships are especially vulnerable: ants, their insect partners and plants can all be affected depending on the strength of the relationship. For example, many species of Lycaenid butterfly depend on ants for their survival (Jordano & Thomas, 1992): climate change effects that result in asynchrony between ants and the butterfly could therefore have detrimental outcomes. But, species that depend heavily, even if not entirely, on another species for nutrition or shelter may also be vulnerable to population declines. The extent to which ants are dependent on particular trophobionts or whether they can easily switch (e.g., diet) is therefore critical if we are to understand fully the consequences of climate change on mutualisms.

Habitat change linked to climate change adds additional complexity to these ant–plant and ant–insect interactions. Examples of habitat change linked to rising atmospheric CO_2_, or increased temperatures and precipitation are now widespread. Vegetation is shifting along both elevational and latitudinal gradients, woody thickening is becoming widespread in open ecosystems, and fires are increasing in severity and frequency in many regions (Barlow et al., 2018); these effects may alter biome integrity with potential switches in ecosystem state and functioning as a consequence. Thus, although many ants may be relatively resilient to climate change, changes that affect nest sites, resource availability and microclimates may impose additional stress. For example, across Africa woody thickening in open savannas and grasslands is increasing because elevated atmospheric CO_2_ favors woody C4 species at the expense of C3 grasses. With the loss of the grassy understory, increased shading and build‐up of a litter layer, Parr et al. (2012) found that not only did species composition of the ant community shift, but there were functional consequences with increases particularly in predatory ant species. Given the widespread nature of encroachment, the potential for the loss of open ecosystem‐associated species (e.g., seed‐harvesters) is a concern. Conversely, in some tropical forested regions, climate has increased habitat flammability with repeated fires driving the transition to degraded forest and open grass‐dominated habitat (Barlow et al., 2018). Studies now indicate that the ant fauna too undergoes a parallel change as loss of forest‐associated species decline while open‐associated epigeic species increase (Bishop et al., 2021; Paolucci et al., 2017).

## 4 HOTSPOTS OF GEOGRAPHIC, PHYLOGENETIC, AND PHENOTYPIC VULNERABILITY

If we can identify which geographic regions, macrohabitats, microhabitats and ant clades are most at risk from climate change, we will be in a better position to predict and understand what the consequences of population declines, and species losses will be. Which are most likely to be affected, but also, which may be able to resist or adapt to climate change?

Although climate models suggest temperature increases at low latitudes will not be as great as those experienced at high latitudes, tropical ants are considered more vulnerable because they generally have lower warming tolerance and already operate closer to their critical thermal limits than temperate ants (Diamond et al., 2012). Globally, therefore, ants in the Tropics at low elevations are most at risk due to their physiological susceptibility (Diamond et al., 2012; Fitzpatrick et al., 2011). Although models vary, overall drying scenarios are anticipated for the eastern Amazon, Central and West African rainforests, Indonesia, and Malaysia by end of 21st century (NOAA): combined with temperature effects, we predict that ants in these regions are likely among the most vulnerable globally. Unfortunately, because these are also the regions that harbor the bulk of ant diversity globally (Dunn et al., 2009; Economo et al., 2018), the consequences of extensive ant declines across the Tropics, combined with widespread disruption of their associated interactions, have the potential to be catastrophic.

**FIGURE 3:**
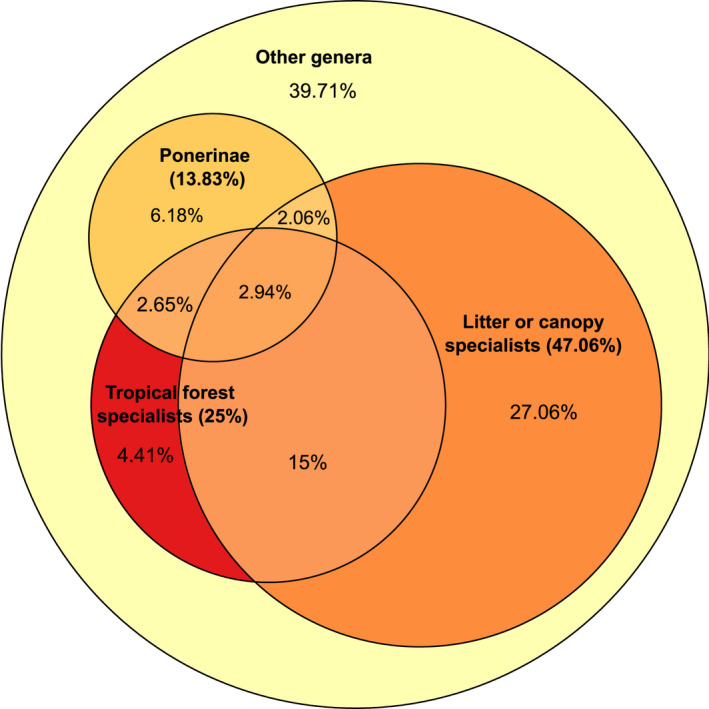
Euler diagram illustrating the percentage of ant genera (*n* = 340) worldwide that are in the at‐risk categories that we identified based on biome, nesting microhabitat and phylogeny. Ant data are based on publicly available global specimen records from AntWeb.org. We extracted specimen metadata to determine whether specimens were collected in the canopy, the leaf litter, or elsewhere. We assigned the most common of these microhabitat classifications within each genus to the entire genus. Geographic coordinates from each specimen were used in combination with the WWF global biome classifications (Olson et al., 2001) to identify genera that were collected in tropical moist broadleaf forest ≥75% of the time. These genera were classed as tropical forest specialists. In the Euler diagram, bold text and percentages refer to each of the primary risk categories (Ponerines, litter or canopy specialists, tropical forest specialists). Percentages in normal text refer to the fractions of genera that fall into two or more of the identified risk categories. For example, 15% of ant genera are both tropical forest and litter or canopy specialists. Only 39.71% of ant genera worldwide do not fall into one of these risk categories

Within tropical regions, however, the type and strength of effect will likely further vary with habitat strata. Plasticity of nesting and brood movement is possible for hypogeic (subterranean) and epigeic (ground‐nesting) ants that live in the thermally buffered soil because temperatures decrease and become more stable with depth, a fact that will likely impact many invertebrate taxa (Duffy et al., 2015). In contrast, canopy and leaf‐litter ants, which have fewer thermal microhabitat refuges available to them, will be particularly vulnerable: they will be more directly exposed to rising temperatures, both inside and outside the nest. The low desiccation resistance of ground ants means that litter ants will be especially susceptible to reduced humidity as a result of reduced precipitation (Hood & Tschinkel, 1990). Across the globe, at least 35.4% of ant genera nest exclusively in the leaf litter and 14.6% exclusively in canopy environments (Lucky et al., 2013; although with a larger sample size we estimate that these values may be much larger, Figure 3). Consequently, for this large fraction of ant diversity a warmer world will likely lead to increased competition for environmentally suitable nesting locations and potentially reduced fitness; common garden experiments have shown that ants brought into suboptimal and higher temperatures have reduced fitness and increased mortality (Pelini et al., 2012), highlighting that if behavioral responses mean ants are unable to source cooler microclimates, there will likely be fitness‐related consequences of warming for ants.

In addition to macro‐ and microhabitat risk factors, there are several traits that will make particular ant taxa more susceptible to climate change. For example, cold climates tend to drive the evolution of larger body size, while smaller body sizes are favored under hot conditions (and, possibly, high CO_2_). Although there are several explanations for the temperature‐size rule (Verberk et al., 2021), this relationship suggests large bodied ants would be at more of a disadvantage with rising temperatures (although see Baudier et al., 2015). Dark coloration and low physiological thermal tolerance of ants are also a disadvantage with increasing temperatures. Most species in the basal ant subfamily Ponerinae are dark colored (indeed many are entirely black), and they tend to have a relatively large body size. Furthermore, thermal tolerances for Ponerine, and large bodied‐species, species tend to be low (typically <42°C, Bishop et al., 2017; Boyle et al., 2021; Penick et al., 2017). Taken together, these traits of body size, color, and thermal tolerance suggest that the Ponerinae may be disproportionately affected by rising temperatures associated with climate change.

When considering the risk factors of biome (tropical rainforest), microhabitat (canopy and litter‐nesting), and phylogeny (Ponerinae), the majority of ant genera (60%) fall into one of these categories, and ~20% into two of more risk categories (Figure 3), suggesting a large proportion of ant species could be negatively affected. For instance, the diverse genera *Hypoponera* and *Pachycondyla* fall into these three risk categories. To calculate these percentages, we extracted georeferenced specimen‐level data for all extant genera from AntWeb.org, assigned each genus crude microhabitat associations by text‐mining their collection metadata, and assigned them biome identities from the WWF (Olson et al., 2001). This is analysis is coarse, but illustrative of these kind of trends. Detailed species‐level analyses are likely to find quantitatively different answers, but we suspect that they will not change the conclusion that a large fraction of ant diversity is at risk of climate change due to their geographic or ecological contexts.

A further factor that will undoubtedly make species more or less vulnerable to climate change is dispersal ability. The ability to track climate envelopes will be critical for many species, but the extent to which that is possible will depend on dispersal ability: species that only disperse short distances (<tens of meters) are therefore expected to be most at risk. These include species that rely on Dependent Colony Foundation (DCF); here the queen and a few nestmate workers leave the original colony and disperse on foot (Peeters, 2012; Peeters & Molet, 2010). In addition, species that use non‐claustral independent colony foundation may also be at risk because the queen must forage outside the nest until the workers are able to takeover this role—to what extent queens can tolerate changing conditions outside the nest is virtually unknown. This strategy is used widely in poneroid species (Peeters & Ito, 2015; Peeters & Molet, 2010) enhancing their risk. To further compound the vulnerability of Ponerines, the queens tend to have low fecundity and consequently colonies are small (Wilson & Hölldobler, 2005). Thus, while Ponerines have been globally successful despite being socially primitive (Hölldobler & Wilson, 2009; Wilson & Hölldobler, 2005), under global climate change this may be their Achilles heel. The potential phylogenetic signal in climate change impacts could have important implications when considering their role in the wider ecosystem because most Ponerines are predators (Boyle et al., 2021; Pfeiffer et al., 2014; Schmidt & Shattuck, 2014). Loss of predators and their trophic control could result in a reorganization of interactions among ants and with their prey (see Section 7). The effects might be particularly acute in Africa given its high species richness of Ponerines (Schmidt & Shattuck, 2014; AntWeb.org, 29 Afrotropical Ponerinae genera, the most of an biogeographic realm). It is also likely that other groups across the phylogeny fit some of these criteria and will also be vulnerable (e.g., some Dorylinae).

Other ants that may be disproportionately affected include nocturnal ants. Temperatures are expected to increase at night as well as in the day, and yet nocturnal ants have much lower thermal tolerance than day—active ants (Garcia‐Robledo et al., 2018)—consequently, nocturnal ants may face significant challenges, particularly in cooler environments. In addition, it is possible day‐active ants may shift their window of activity to cooler periods of the day (dusk, evening) and may possibly outcompete night‐active ants. Day‐active ants could shift foraging to become crepuscular or nocturnal if the ants do not rely heavily on visual cues (Cros et al., 1997; Greenaway, 1981; Houadria & Menzel, 2020; Jayatilaka et al., 2011) and are not constrained by availability of resources and competition or predation risk. This remains to be determined, however.

The focus here has been on ants most at risk and yet, there are a number of ants that will likely do much better with climate change. Ants in temperate regions will benefit from increased temperatures with positive effects on development and foraging. In addition, the majority of ants in these regions are soil‐nesting and can therefore take advantage of the buffering soil provides. While Ponerine predators may be particularly at risk, herbivorous ants, such as the Formicines, which dominate temperate regions, may be able to take advantage of increases in honeydew availability predicted under climate change and increase in abundance. Overall, we predict a shift in trophic dynamics and the dominance of ants at lower trophic levels. To what extent these shifts may cascade through the wider food‐web is uncertain but given that ants not only decrease predation and parasitism of honeydew‐producing Hemiperans but also decrease survival and abundance of other insect herbivores (Styrsky & Eubanks, 2007), it is plausible that overall plant herbivores may decline with cascading consequences across the wider food‐web.

## 5 THE SOCIAL DIMENSION

The eusocial nature of ants often goes unappreciated in work attempting to understand their current and future responses to the environment. Eusociality, however, may strongly modulate how ants respond to climate change. Solitary organisms must complete tasks sequentially (i.e., nest building, then egg laying, then foraging, then larvae provisioning), but eusocial organisms, like ants, can perform these tasks in series‐parallel (Wilson, 1990), that is, ant colonies can perform each of these different tasks simultaneously. This is because multiple workers attempt each task at the same time and can move flexibly between them as the need arises. This series‐parallel processing contributes to two key ecological advantages that we argue are likely offer ants an advantage relative to solitary insects and ectotherms under climate change.

First, ant colonies can maintain fine homeostatic control over their nest environments. Series‐parallel behavior means that many ants can control their nesting environments constantly, without sacrificing the need to reproduce, forage, or defend themselves. Ants can actively and passively regulate temperature (Jones & Oldroyd, 2006), humidity (Bollazzi & Roces, 2010), and CO_2_ levels (Bollazzi & Roces, 2007). This means that they can often find optimal conditions for growth and survival in environments that otherwise appear inhospitable. For instance, the Floridian winter ant, *Prenolepis imparis*, moves its active nesting chambers up and down the soil column depending on the season—different depths provide different temperatures (Tschinkel, 1987). As we discuss above, ants can also move their nests entirely to find more optimal thermal conditions (McGlynn et al., 2010) and can move their brood within the nest to optimize their developmental rates (Penick & Tschinkel, 2008). For those species that have access to these kinds of natural thermal gradients, finding lower nesting temperatures as the Earth warms may be a relatively easy behavioral adaptation. This offers ants a potentially large advantage compared with solitary organisms who must otherwise compete among themselves for the fewer remaining cool microsites within which to nest and reproduce. Of course, as we mention above, this effect is not likely to be uniformly available across the environments that ants live in. In rainforest canopies, for instance, there may be fewer and shallower thermal gradients down which ant colonies can retreat and maintain optimal nest homeostasis.

**FIGURE 4:**
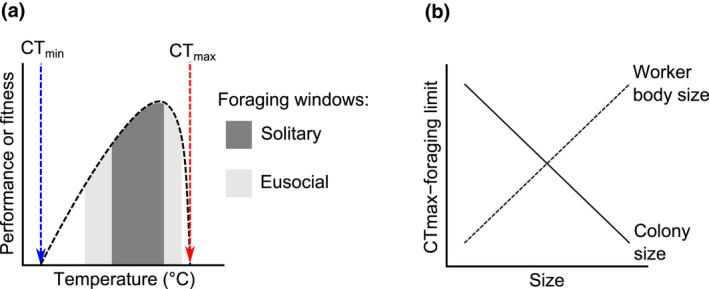
Predictions of how sociality interacts with thermal tolerance. In (a) we predict that the thermal foraging windows of eusocial species will be wider than those of solitary species, with foraging limits being closer to the critical temperatures (CT_min_ or CT_max_, dashed vertical lines). The diagram shows a typical thermal performance curve (black dashed curve). In (b) we predict that both colony size and individual body size will determine the amount of risk taken by social animals in the light of climate change. Colony or body size is represented on the x‐axis. The difference between the organism's CT_max_ and the highest temperature at which they forage is represented on the y‐axis. Organisms foraging close to their CT_max_ will appear lower on the y‐axis. We predict that larger colonies will forage closer to their CT_max_ as individual workers will be more expendable. Furthermore, we predict that smaller workers will forage closer to their CT_max_ because they can more easily thermoregulate due to the low thermal inertia offered by their small size—small organisms heat up quickly but similarly cool down quickly once in the shade

Second, worker ants are expendable. The relationship between individual ants and the colony is analogous to that between a single cell and the body of a solitary organism (Hölldobler & Wilson, 2009). Consequently, the loss of a worker in the defence of the nest, or on a foraging trip under lethal conditions, will not cause the collapse of the entire colony. Series‐parallel processing ensures that there is another worker to pick up and complete the task. This is not the case for solitary organisms. For them, death while foraging or defending a resource means that they can no longer continue to contribute to the next generation. Given this, we expect that ants and other eusocial animals will be able to achieve very different risk‐reward ratios than solitary organisms as the climate changes. For instance, ants may be able to afford to forage much closer to their thermal limits than solitary organisms (Figure 4a). In hot environments, ants are known to forage close to their maximum thermal tolerances, when no other animal can forage. They routinely climb up vegetation or seek shade to cool down before continuing to search for food (Shi et al., 2015; Wehner & Wehner, 2011). Consequently, we predict that social organisms will be better able to absorb the risks of living under higher temperature regimes than solitary organisms. This argument is based on first principles, however, and it remains to be seen whether this effect plays out empirically. For instance, Mediterranean ants may buck this trend (Arnan et al., 2012), as might invasive ants outside of their native range as they develop large colony sizes without foraging at the highest temperatures (Angulo et al., 2011). It is unknown what that consequences of this prediction may be on social and non‐social population dynamics—there are likely to be interactions, for example, with body size and colony size (Figure 4b). We predict that larger colonies would be able to take on more risk, and forage closer to their CT_max_. Similarly, we predict that species with small workers, which gain and lose heat rapidly, will be able to forage closer to their thermal limits. However, differences in relative leg length may allow some species to stilt above the superheated boundary layer of air sitting atop walkable surfaces (Kaspari et al., 2015; Sommer & Wehner, 2012). This morphological shape difference may alter the outcomes of the simple size‐foraging limit relationships we pose here.

These two effects, homeostasis and worker expendability, are likely to allow ants to ride out environmental changes to a much greater degree than solitary organisms. This may further tilt the dominance of Earth's terrestrial systems in favor of eusocial insects like ants (Tuma et al., 2020). A greater understanding is needed, however, of how these effects may influence survival, growth, and population stability among and between social and solitary animals.

## 6 ADAPTATION, PLASTICITY, AND DISPERSAL

A key maxim of climate change biology is that organisms must adapt, move, or die (Donoghue, 2008; Pecl et al., 2017). As temperatures rise and the climate changes, organisms could opt to disperse and track the conditions to which they are already adapted—typically toward higher elevations and latitudes. This appears to be the default option for many organisms and is already visible in the ongoing movement and reorganization of ecological communities across the planet (Chen et al., 2011; Pecl et al., 2017). It seems that in many cases it is easier to move than to evolve (Donoghue, 2008). Alternatively, species can adapt to their new conditions in two ways. They may genetically adapt via natural selection to cope with their new environment, or they may adapt plastically by exploiting existing behavioral and physiological variability (Merilä & Hendry, 2014). Understanding which of these options are available to different ant species is key to understanding their response to climate change. Data to assess the likelihood of these alternative scenarios, however, is severely lacking.

For instance, in terms of ant dispersal, most available data are summarized in a recent review of ant flight ecology (Helms, 2018). These data show that queen ants can fly between a few meters to around 30 km from their mother‐nests, and that this variation is driven partly by body size: larger queens tend fly further. The phylogenetic coverage of these data, however, is poor. Almost no data exist on the basal Ponerinae group (Helms, 2018), and the remaining data capture only a tiny fraction of known ant diversity (16 species vs. ~30,000 ant species estimated to exist). Consequently, it is unclear how far ants may typically be able to disperse. Taking these estimates at face value, however, shows that ants which disperse on the wing may be able to travel ~6 km on average per dispersal event (mean = 7.4 km, median = 5.7 km). Assuming annual dispersal events, this value of 6 km is above the 1.6 km per year average rate of movement recorded for a range of taxonomic groups in response to climate change (Chen et al., 2011). This suggests that, in theory, and on average, ants will be able to disperse fast enough to keep pace with their existing climatic niches and tolerances. The key questions, however, surround the variability of ant dispersal, whether this variation is phylogenetically biased, and to what extent species are differently constrained in being able to establish following dispersal into new geographic regions. Further, these data do not take into account the poor dispersal exhibited by species that do not fly. Most army ants (subfamily Dorylinae), for example, and numerous other species across the ant phylogeny, found new colonies via Dependent Colony Foundation (DCF) (Section 4). It is not clear how far ants reliant on DCF will be able to disperse, but we expect that there will be a much lower upper limit in comparison with species, which disperse via flight and a much greater impact of barriers such as rivers (Soare et al., 2014).

Data on the adaptive response of ants to climate change is also scant. Reciprocal transplant experiments have shown how acorn ants (*Temnothorax nylanderi*) adapt to their local thermal environment. Martin et al. (2021) transplanted acorn ant colonies living in urban environments into their ancestral rural environment, and vice versa. They found strong evidence that the thermal tolerances of these groups were locally adapted—the urban populations had higher maximum and minimum thermal tolerances—and that these adaptations contributed to higher survival in their home versus their novel environment. The authors ruled out the influence of physiological plasticity in this case because subsequent generations of lab‐reared colonies also displayed divergence in their thermal tolerances (Martin et al., 2019). While this adaptive pattern was found across multiple, independent rural‐urban gradients in the United States (Diamond et al., 2017, 2018), it is unclear whether ants in general will respond in this way to thermal changes and over which timescales. On the other hand, ants also seem capable of plastically altering their phenotype in response to environmental changes. In the same acorn ant system, thermal tolerance could also plastically respond to rearing temperature, with higher rearing temperatures leading to individuals with higher thermal tolerances (Diamond et al., 2017). Similarly, Bujan et al. (2020) found evidence for seasonal plasticity in thermal tolerance across a number of other American ant species. Workers collected in the summer had higher thermal tolerances than those collected during cooler months. Again, how widespread these phenomena are is currently unclear, but combined with dispersal provide a mechanism by which ants can respond to climate change alongside their great behavioral flexibility (discussed above).

## 7 ECOSYSTEM CONSEQUENCES

The direct and indirect effects of climate change are both likely to influence the structure of local ant communities and their contribution to key ecosystem functions. Ants are considered to play important roles in ecosystem functioning via their physical activities (influencing soil in particular) and trophic interactions (e.g., predation, seed harvesting/dispersal) (Del Toro et al., 2012; Folgarait, 1998). Given their high biomass and abundance in most ecosystems, declines or changes to ant community structure have the potential for large impacts on the wider ecosystem. There have been few studies directly exploring the implications of climate change for ecosystem functions and services provided by ants. In part, understanding how ecosystems will change with fewer, or different ants, is challenging because there have been relatively few studies examining the contribution that ants alone make, particularly at an ecosystem level. Here we consider the ecological processes and functions ants contribute to and explore how this might change with altered ant abundance.

Bioturbation, the reworking of soil by ants through movement, tunneling and nest‐building activities, is an essential ecosystem service. This process can profoundly affect nutrient distribution (e.g., increase P concentration in ant nests) and soil physical properties including soil porosity (Dostál et al., 2005; Frouz et al., 2003; Nkem et al., 2000) and promotes plant biodiversity (Wilkinson et al., 2009) and growth (Reichman & Seabloom, 2002). Ants also redistribute and alter nutrient availability within ecosystems through consumption of plant material, detritus and seeds combined with bioturbation. Ants can play a critical role as scavengers in tropical rainforest (Griffiths et al., 2018), and importantly, when ants were excluded there was no compensation by other taxa suggesting low functional redundancy if ant numbers decline. In urban areas too ants can consume and recycle human food inputs, and therefore play a role in waste removal, with invasive ants especially important (Penick et al., 2015; Youngsteadt et al., 2015).

Many ants are seed harvesters and through seed predation can strongly influence plant composition and diversity where they occur (De Almeida et al., 2020; MacMahon et al., 2000). For many processes, it is not known how important ants are relative to other taxa (e.g., rodents) because experiments that manipulate different groups are needed to disentangle contributions. A long‐term manipulative experiment at Portal, Arizona, however, found that rodent and harvester ants compete for food, and ants increased plant diversity by differentially harvesting seeds of dominant species (Brown et al., 1979).

As competitors and predators, ants are thought to strongly shape communities of plants and animals. Manipulative experiments have demonstrated powerfully that the presence of ants alters invertebrate community structure and food webs (Parr et al., 2016), with some suggestion that herbivores and decomposers do better when ant abundances are low (Sanders & van Veen, 2011). Similarly, low ant abundance can promote the dominant decomposer invertebrate in savannas, termites, with positive effects on decomposition (Parr et al., 2016). Given that predatory ants, including Ponerines, may be particularly susceptible to climate change (Section 4 above) and there may be a change in trophic structure, predation pressure may decline in future with important implications for prey (e.g., termites) and consequently the processes the prey mediates (e.g., decomposition). Broad changes in ant community composition are highly likely (Bishop et al., 2019) and the dominant ant species may alter too. This matters because we know dominant ants exert considerable pressure on local communities and can regulate community structure via competition and predation (Andersen & Patel, 1994; Parr, 2008). It is possible under climate change that there will be a shift in the dominance of ants globally from the Tropics to temperate regions as tropical ants fare less well and those at higher latitudes are able to take advantage; the high number of ground‐nesting and herbivorous ants in temperate regions also lends further support to this idea.

The value of ants for pest‐control has long been recognized as ants are voracious predators of plant herbivores and can reduce herbivory (Hölldobler & Wilson, 1990), but only more recently have studies partitioned out the value of ants versus other taxa; for example, Piñol et al. (2010) demonstrated that ants had a greater effect regulating arthropod populations on citrus than birds did. The predation biocontrol service provided by ants is especially valued in agricultural systems with studies suggesting ants can significantly promote crop yields (e.g., in shade cocoa, Gras et al., 2016). In addition, ant pest‐control services extend to weed control too: in an Australian agricultural system, the presence of ants reduced the number of tumbleweed plants by almost a half (Evans & Gleeson, 2016). Predicted declines in arboreal ants in the Tropics will therefore be especially detrimental to agriculture in these regions.

Finally, disruptions to mutualisms will have important ramifications across ecosystems. In particular, where seed dispersal by ants (myrmecochory) declines due to phenological mismatches between plant species and their disperser ants, or fewer ants to disperse seeds, there will likely be shifts in the composition of plant communities; the breakdown of seed dispersal mutualism in the Fynbos with the introduction of the invasive Argentine ant demonstrates this potential outcome clearly (Christian, 2001). Similarly, there will also be community‐level implications for arthropod communities with changes to the relationship between ants and honeydew‐dew producing insects (Hemipterans). Because these ants often also predate on other plant herbivores (Kaplan & Eubanks, 2005), they can dramatically alter the structure of the arthropod community. This has knock‐on effects for plant protection services as reduced non‐Hemipteran herbivory damage can result in increased plant growth and seed production (Styrsky & Eubanks, 2007). While the negative effects of interactions between ants and Hemipterans on plant fitness have seldom been demonstrated, where there is a strong positive feedback between Hemipterans and ants, there can be detrimental outcomes for plants (e.g., the association between Yellow crazy ants and scale insects on Christmas Island resulted in the growth of sooty mould on leaves and canopy die‐back, O'Dowd et al., 2003). How climate change‐induced changes to ants will ultimately affect primary production is unknown.

## 8 KNOWLEDGE GAPS AND FUTURE QUESTIONS

We identify several key research knowledge gaps and testable hypotheses which we argue are critical to address for us to understand more fully the implications of a changing climate on the ants. These include:

1. How will altered precipitation, CO_2_ and UV regimes affect ants? Currently, we know very little about these important drivers. And, because climate change drivers do not operate in isolation, it is essential we explore how they may, or may not, interact with temperature change.
2. How will variable ant performance at sublethal temperatures play out in altered thermal regimes? It is becoming clear that critical temperatures do not explain all the variation in ant distribution and activity (Braschler et al., 2020; Guo et al., 2020), yet ecological performance at sublethal temperatures, and how this impacts individual fitness and population growth, is vastly understudied.
3. How does sociality alter the risk‐reward ratios of foraging under altered thermal regimes? We hypothesize here that social organisms will respond differently to solitary organisms, and that this response may depend on body and colony size (Section 5, Figure 4). Empirical data testing this, and accounting for other morphological traits such as leg length (particularly for ground‐active taxa), will be key to understanding potential changes to relative success of solitary and social organisms under climate change.
4. How do ants disperse and establish new nests? How do these processes feed into population dynamics? There is extremely limited fundamental empirical data on these topics within the ants, yet they are critical in fully predicting the response of ants to climate change.
5. What are the species‐specific contributions to ecosystem functioning? We are moving towards a better understanding of how ants in general influence the ecosystem (e.g., Parr et al., 2016), but because there will be differential effects of climate change on ants, we need to know more about how individual species contribute. Only when we know this can we understand what the ecosystem consequences will be of altered and rearranged ant diversity. The wider impact of altered or disrupted mutualisms also falls into this area.
6. Finally, we need a shift in the focus for much of this work to the Tropics—this is where effects are likely to be most pronounced, and it is especially urgent we understand how ant communities will respond and what the implications will be.

## 9 CONCLUSION

In sum, we face huge knowledge gaps in terms of our understanding of how ants will respond to climate change. We know the most about the thermal response of ants. In this case, we expect that Tropical species, particularly those living in the litter of canopy microhabitats, will be more vulnerable to rising temperatures. Elsewhere in the world, and for those species, which can exploit their eusociality behaviorally to avoid increased temperatures for large parts of their developmental and foraging activities, we anticipate minimal impacts or even benefits. It is far from clear how ants will respond to altered precipitation, CO_2_, or UV regimes. Furthermore, how these climatic changes will ramify through the complex food and mutualistic webs within which ants are embedded are largely unknown, but we anticipate that they may be profound. What does seem clear, based on the phylogenetic signal of ecologies and traits among the ants, is that the Ponerinae appear particularly vulnerable to changing climates. More broadly, we expect that there will a rearrangement of the current patterns of ant diversity and community structures. It is these changes that we urge a focus on, particularly in the context of ecosystem function, only then we can predict and begin to mitigate the climatic impacts on this important group of insects.

## Data Availability

Data sharing not applicable as no new data created or analysed.

## References

[gcb16140-bib-0001] Almeida, R. P. , Silva, R. R. , da Costa, A. C. , Ferreira, L. V. , Meir, P. , & Ellison, A. M. (2020). Induced drought strongly affects richness and composition of ground‐dwelling ants in the eastern Amazon. bioRxiv. 10.1007/s00442-023-05316-x 36645473

[gcb16140-bib-0002] Andersen, A. N. (1983). Species diversity and temporal distribution of ants in the semi‐arid mallee region of northwestern Victoria. Australian Journal of Ecology, 8(2), 127–137. 10.1111/j.1442-9993.1983.tb01600.x

[gcb16140-bib-0003] Andersen, A. N. , & Patel, A. (1994). Meat ants as dominant members of Australian ant communities: An experimental test of their influence on the foraging success and forager abundance of other species. Oecologia, 98(1), 15–24. 10.1007/BF00326085 28312791

[gcb16140-bib-0004] Angilletta, M. J. (2009). Thermal adaptation: A theoretical and empirical synthesis. Oxford University Press.

[gcb16140-bib-0005] Angulo, E. , Caut, S. , & Cerdá, X. (2011). Scavenging in Mediterranean ecosystems: Effect of the invasive Argentine ant. Biological Invasions, 13(5), 1183–1194. 10.1007/s10530-011-9953-6

[gcb16140-bib-0006] Archibald, S. B. , Johnson, K. R. , Mathewes, R. W. , & Greenwood, D. R. (2011). Intercontinental dispersal of giant thermophilic ants across the Arctic during early Eocene hyperthermals. Proceedings of the Royal Society B: Biological Sciences, 278(1725), 3679–3686. 10.1098/rspb.2011.0729 PMC3203508 21543354

[gcb16140-bib-0007] Arnan, X. , Cerdá, X. , & Retana, J. (2012). Distinctive life traits and distribution along environmental gradients of dominant and subordinate Mediterranean ant species. Oecologia, 170(2), 489–500. 10.1007/s00442-012-2315-y 22476711

[gcb16140-bib-0008] Arnan, X. , Silva, C. H. F. , Reis, D. Q. A. , Oliveira, F. M. P. , Câmara, T. , Ribeiro, E. M. S. , Andersen, A. N. , & Leal, I. R. (2022). Individual and interactive effects of chronic anthropogenic disturbance and rainfall on taxonomic, functional and phylogenetic composition and diversity of extrafloral nectary‐bearing plants in Brazilian caatinga. Oecologia, 198, 267–277. 10.1007/s00442-021-05074-8 34767071

[gcb16140-bib-0009] Banschbach, V. , Levit, N. , & Herbers, J. (1997). Nest temperatures and thermal preferences of a forest ant species: Is seasonal polydomy a thermoregulatory mechanism? Insectes Sociaux, 44(2), 109–122. 10.1007/s000400050034

[gcb16140-bib-0010] Barlow, J. , França, F. , Gardner, T. A. , Hicks, C. C. , Lennox, G. D. , Berenguer, E. , & Guénard, B. (2018). The future of hyperdiverse tropical ecosystems. Nature, 559(7715), 517–526. 30046075 10.1038/s41586-018-0301-1

[gcb16140-bib-0011] Barton, B. T. , & Ives, A. R. (2014). Direct and indirect effects of warming on aphids, their predators, and ant mutualists. Ecology, 95(6), 1479–1484. 10.1890/13-1977.1 25039213

[gcb16140-bib-0012] Bates, O. K. , Ollier, S. , & Bertelsmeier, C. (2020). Smaller climatic niche shifts in invasive than non‐invasive alien ant species. Nature Communications, 11(1), 1–8. 10.1038/s41467-020-19031-1 PMC7567077 33060612

[gcb16140-bib-0013] Baudier, K. M. , Mudd, A. E. , Erickson, S. C. , & O'Donnell, S. (2015). Microhabitat and body size effects on heat tolerance: implications for responses to climate change (army ants: Formicidae, Ecitoninae). Journal of Animal Ecology, 84(5), 1322–1330. 10.1111/1365-2656.12388 26072696

[gcb16140-bib-0014] Beattie, A. J. , & Hughes, L. (2002). Ant–plant interactions. In C. M. Herrera , & O. Pellmyr (Eds.), Plant‐animal interactions and evolutionary approach (pp. 211–235). Blackwell Science.

[gcb16140-bib-0015] Bellard, C. , Bertelsmeier, C. , Leadley, P. , Thuiller, W. , & Courchamp, F. (2012). Impacts of climate change on the future of biodiversity. Ecology Letters, 15(4), 365–377. 10.1111/j.1461-0248.2011.01736.x 22257223 PMC3880584

[gcb16140-bib-0016] Berman, D. I. , Alfimov, A. V. , Zhigulskaya, Z. A. , & Leirikh, A. N. (2010). Overwintering and cold hardiness of ants in the northeast of Asia. Pensoft.

[gcb16140-bib-0017] Bernstein, R. A. (1979). Schedules of foraging activity in species of ants. Journal of Animal Ecology, 48, 921–930. 10.2307/4204

[gcb16140-bib-0018] Bertelsmeier, C. , Blight, O. , & Courchamp, F. (2016). Invasions of ants (Hymenoptera: Formicidae) in light of global climate change. Myrmecological News, 22, 25–42.

[gcb16140-bib-0019] Bertelsmeier, C. , Luque, G. M. , Hoffmann, B. D. , & Courchamp, F. (2015). Worldwide ant invasions under climate change. Biodiversity and Conservation, 24(1), 117–128. 10.1007/s10531-014-0794-3

[gcb16140-bib-0020] Bishop, T. R. , Parr, C. L. , Gibb, H. , Rensburg, B. J. , Braschler, B. , Chown, S. L. , Foord, S. H. , Lamy, K. , Munyai, T. C. , Okey, I. , Tshivhandekano, P. G. , Werenkraut, V. , & Robertson, M. P. (2019). Thermoregulatory traits combine with range shifts to alter the future of montane ant assemblages. Global Change Biology, 25(6), 2162–2173. 10.1111/gcb.14622 30887614

[gcb16140-bib-0021] Bishop, T. R. , Robertson, M. P. , Gibb, H. , van Rensburg, B. J. , Braschler, B. , Chown, S. L. , Foord, S. H. , Munyai, T. C. , Okey, I. , Tshivhandekano, P. G. , Werenkraut, V. , Parr, C. L. , & Pincheira‐Donoso, D. (2016). Ant assemblages have darker and larger members in cold environments. Global Ecology and Biogeography, 25, 1489–1499. 10.1111/geb.12516

[gcb16140-bib-0022] Bishop, T. R. , Robertson, M. P. , van Rensburg, B. J. , & Parr, C. L. (2014). Elevation–diversity patterns through space and time: Ant communities of the Maloti‐Drakensberg Mountains of southern Africa. Journal of Biogeography, 41(12), 2256–2268. 10.1111/jbi.12368

[gcb16140-bib-0023] Bishop, T. R. , Robertson, M. P. , Van Rensburg, B. J. , & Parr, C. L. (2017). Coping with the cold: minimum temperatures and thermal tolerances dominate the ecology of mountain ants. Ecological Entomology, 42(2), 105–114. 10.1111/een.12364

[gcb16140-bib-0024] Bishop, T. R. , Tomlinson, A. , McNeice, T. , Sfenthourakis, S. , & Parr, C. L. (2021). The effect of fire on ant assemblages does not depend on habitat openness but does select for large, gracile predators. Ecosphere, 12(6), e03549. 10.1002/ecs2.3549

[gcb16140-bib-0025] Blanchard, S. , Lognay, G. , Verheggen, F. , & Detrain, C. (2019). Today and tomorrow: impact of climate change on aphid biology and potential consequences on their mutualism with ants. Physiological Entomology, 44(2), 77–86. 10.1111/phen.12275

[gcb16140-bib-0026] Bollazzi, M. , Kronenbitter, J. , & Roces, F. (2008). Soil temperature, digging behaviour, and the adaptive value of nest depth in South American species of Acromyrmex leaf‐cutting ants. Oecologia, 158(1), 165–175. 10.1007/s00442-008-1113-z 18668265

[gcb16140-bib-0027] Bollazzi, M. , & Roces, F. (2007). To build or not to build: Circulating dry air organizes collective building for climate control in the leaf‐cutting ant Acromyrmex ambiguus. Animal Behaviour, 74(5), 1349–1355. 10.1016/j.anbehav.2007.02.021

[gcb16140-bib-0028] Bollazzi, M. , & Roces, F. (2010). Leaf‐cutting ant workers (Acromyrmex heyeri) trade off nest thermoregulation for humidity control. Journal of Ethology, 28(2), 399–403. 10.1007/s10164-010-0207-3

[gcb16140-bib-0029] Boyle, M. J. W. , Bishop, T. R. , Luke, S. H. , Breugel, M. , Evans, T. A. , Pfeifer, M. , Fayle, T. M. , Hardwick, S. R. , Lane‐Shaw, R. I. , Yusah, K. M. , Ashford, I. C. R. , Ashford, O. S. , Garnett, E. , Turner, E. C. , Wilkinson, C. L. , Chung, A. Y. C. , & Ewers, R. M. (2021). Localised climate change defines ant communities in human‐modified tropical landscapes. Functional Ecology, 35(5), 1094–1108. 10.1111/1365-2435.13737

[gcb16140-bib-0030] Braschler, B. , Duffy, G. A. , Nortje, E. , Kritzinger‐Klopper, S. , Plessis, D. , Karenyi, N. , Leihy, R. I. , & Chown, S. L. (2020). Realised rather than fundamental thermal niches predict site occupancy: Implications for climate change forecasting. Journal of Animal Ecology, 89(12), 2863–2875. 10.1111/1365-2656.13358 32981063

[gcb16140-bib-0031] Brian, M. , & Brian, A. (1951). Insolation and ant population in the west of Scotland. Transactions of the Royal Entomological Society of London, 102(6), 303–330. 10.1111/j.1365-2311.1951.tb00751.x

[gcb16140-bib-0032] Brown, J. H. , Davidson, D. W. , & Reichman, O. (1979). An experimental study of competition between seed‐eating desert rodents and ants. American Zoologist, 19(4), 1129–1143. 10.1093/icb/19.4.1129

[gcb16140-bib-0033] Bujan, J. , Roeder, K. A. , Yanoviak, S. P. , & Kaspari, M. (2020). Seasonal plasticity of thermal tolerance in ants. Ecology, 101, e03051. 32239508 10.1002/ecy.3051

[gcb16140-bib-0034] Bujan, J. , Yanoviak, S. P. , & Kaspari, M. (2016). Desiccation resistance in tropical insects: causes and mechanisms underlying variability in a Panama ant community. Ecology and Evolution, 6(17), 6282–6291. 10.1002/ece3.2355 27648242 PMC5016648

[gcb16140-bib-0035] Carlson, D. M. , & Gentry, J. B. (1973). Effects of shading on the migratory behavior of the Florida harvestor ant. Pogonomyremex Badius. Ecology, 54(2), 452–453. 10.2307/1934357

[gcb16140-bib-0036] Cerdá, X. , Retana, J. , & Cros, S. (1998). Critical thermal limits in Mediterranean ant species: Trade‐off between mortality risk and foraging performance. Functional Ecology, 12(1), 45–55. 10.1046/j.1365-2435.1998.00160.x

[gcb16140-bib-0037] Chen, I.‐C. , Hill, J. K. , Ohlemüller, R. , Roy, D. B. , & Thomas, C. D. (2011). Rapid range shifts of species associated with high levels of climate warming. Science, 333(6045), 1024–1026. 21852500 10.1126/science.1206432

[gcb16140-bib-0038] Christian, C. E. (2001). Consequences of a biological invasion reveal the importance of mutualism for plant communities. Nature, 413(6856), 635–639. 11675787 10.1038/35098093

[gcb16140-bib-0039] Coley, P. D. , Bryant, J. P. , & Chapin, F. S. (1985). Resource availability and plant antiherbivore defense. Science, 230(4728), 895–899. 17739203 10.1126/science.230.4728.895

[gcb16140-bib-0040] Cros, S. , Cerdá, X. , & Retana, J. (1997). Spatial and temporal variations in the activity patterns of Mediterranean ant communities. Ecoscience, 4(3), 269–278. 10.1080/11956860.1997.11682405

[gcb16140-bib-0041] De Almeida, T. , Blight, O. , Mesléard, F. , Bulot, A. , Provost, E. , & Dutoit, T. (2020). Harvester ants as ecological engineers for Mediterranean grassland restoration: Impacts on soil and vegetation. Biological Conservation, 245, 108547. 10.1016/j.biocon.2020.108547

[gcb16140-bib-0042] Dean, W. , & Turner, J. (1991). Ants nesting under stones in the semi‐arid Karoo, South Africa: Predator avoidance or temperature benefits? Journal of Arid Environments, 21(1), 59–69. 10.1016/S0140-1963(18)30728-6

[gcb16140-bib-0043] Del Toro, I. , Ribbons, R. R. , & Pelini, S. L. (2012). The little things that run the world revisited: A review of ant‐mediated ecosystem services and disservices (Hymenoptera: Formicidae). Myrmecological News, 17, 133–146.

[gcb16140-bib-0044] Delhey, K. (2017). Darker where cold and wet: Australian birds follow their own version of Gloger's rule. Ecography, 41, 673–683. 10.1111/ecog.03040

[gcb16140-bib-0045] Diamond, S. E. , Chick, L. , Perez, A. , Strickler, S. A. , & Martin, R. A. (2017). Rapid evolution of ant thermal tolerance across an urban‐rural temperature cline. Biological Journal of the Linnean Society, 121(2), 248–257. 10.1093/biolinnean/blw047

[gcb16140-bib-0046] Diamond, S. E. , Chick, L. D. , Perez, A. , Strickler, S. A. , & Martin, R. A. (2018). Evolution of thermal tolerance and its fitness consequences: Parallel and non‐parallel responses to urban heat islands across three cities. Proceedings of the Royal Society B: Biological Sciences, 285(1882), 20180036. 10.1098/rspb.2018.0036 PMC6053939 30051828

[gcb16140-bib-0047] Diamond, S. , Nichols, L. , Pelini, S. , Penick, C. , Barber, G. , Cahan, S. , & Gotelli, N. (2016). Climate warming destabilizes forest ant communities. Science Advances, 2, e1600842. 27819044 10.1126/sciadv.1600842 PMC5091351

[gcb16140-bib-0048] Diamond, S. E. , Sorger, D. M. , Hulcr, J. , Pelini, S. L. , Toro, I. D. , Hirsch, C. , Oberg, E. , & Dunn, R. R. (2012). Who likes it hot? A global analysis of the climatic, ecological, and evolutionary determinants of warming tolerance in ants. Global Change Biology, 18(2), 448–456. 10.1111/j.1365-2486.2011.02542.x

[gcb16140-bib-0049] Donoghue, M. J. (2008). A phylogenetic perspective on the distribution of plant diversity. Proceedings of the National Academy of Sciences, 105(Suppl 1), 11549–11555. 10.1073/pnas.0801962105 PMC2556411 18695216

[gcb16140-bib-0050] Dostál, P. , Březnová, M. , Kozlíčková, V. , Herben, T. , & Kovář, P. (2005). Ant‐induced soil modification and its effect on plant below‐ground biomass. Pedobiologia, 49(2), 127–137. 10.1016/j.pedobi.2004.09.004

[gcb16140-bib-0051] Duffy, G. A. , Coetzee, B. W. , Janion‐Scheepers, C. , & Chown, S. L. (2015). Microclimate‐based macrophysiology: Implications for insects in a warming world. Current Opinion in Insect Science, 11, 84–89. 10.1016/j.cois.2015.09.013 28285764

[gcb16140-bib-0052] Dunn, R. R. , Agosti, D. , Andersen, A. N. , Arnan, X. , Bruhl, C. A. , Cerdá, X. , Ellison, A. M. , Fisher, B. L. , Fitzpatrick, M. C. , Gibb, H. , Gotelli, N. J. , Gove, A. D. , Guenard, B. , Janda, M. , Kaspari, M. , Laurent, E. J. , Lessard, J.‐P. , Longino, J. T. , Majer, J. D. , … Sanders, N. J. (2009). Climatic drivers of hemispheric asymmetry in global patterns of ant species richness. Ecology Letters, 12(4), 324–333. 10.1111/j.1461-0248.2009.01291.x 19292793

[gcb16140-bib-0053] Economo, E. P. , Narula, N. , Friedman, N. R. , Weiser, M. D. , & Guénard, B. (2018). Macroecology and macroevolution of the latitudinal diversity gradient in ants. Nature Communications, 9(1), 1–8. 10.1038/s41467-018-04218-4 PMC5934361 29725049

[gcb16140-bib-0054] Evans, T. A. , & Gleeson, P. V. (2016). Direct measurement of ant predation of weed seeds in wheat cropping. Journal of Applied Ecology, 53(4), 1177–1185. 10.1111/1365-2664.12640

[gcb16140-bib-0055] Fabian, B. , Atwell, B. J. , & Hughes, L. (2018). Response of extrafloral nectar production to elevated atmospheric carbon dioxide. Australian Journal of Botany, 66(7), 479–488. 10.1071/BT18012

[gcb16140-bib-0056] Fan, Y. , & Wernegreen, J. J. (2013). Can't take the heat: High temperature depletes bacterial endosymbionts of ants. Microbial Ecology, 66(3), 727–733. 10.1007/s00248-013-0264-6 23872930 PMC3905736

[gcb16140-bib-0057] Fitzpatrick, M. C. , Sanders, N. J. , Ferrier, S. , Longino, J. T. , Weiser, M. D. , & Dunn, R. (2011). Forecasting the future of biodiversity: A test of single‐and multi‐species models for ants in North America. Ecography, 34(5), 836–847. 10.1111/j.1600-0587.2011.06653.x

[gcb16140-bib-0058] Folgarait, P. J. (1998). Ant biodiversity and its relationship to ecosystem functioning: A review. Biodiversity and Conservation, 7(9), 1221–1244. 10.1023/a:1008891901953

[gcb16140-bib-0059] Frouz, J. , Holec, M. , & Kalčík, J. (2003). The effect of Lasius niger (Hymenoptera, Formicidae) ant nest on selected soil chemical properties. Pedobiologia, 47(3), 205–212. 10.1078/0031-4056-00184

[gcb16140-bib-0060] Garcia‐Robledo, C. , Chuquillanqui, H. , Kuprewicz, E. K. , & Escobar‐Sarria, F. (2018). Lower thermal tolerance in nocturnal than in diurnal ants: A challenge for nocturnal ectotherms facing global warming. Ecological Entomology, 43(2), 162–167. 10.1111/een.12481

[gcb16140-bib-0061] Gaudard, C. A. , Robertson, M. P. , & Bishop, T. R. (2019). Low levels of intraspecific trait variation in a keystone invertebrate group. Oecologia, 4, 725–735. 10.1007/s00442-019-04426-9 PMC6704090 31172253

[gcb16140-bib-0062] Gibb, H. , Grossman, B. F. , Dickman, C. R. , Decker, O. , & Wardle, G. M. (2019). Long‐term responses of desert ant assemblages to climate. Journal of Animal Ecology, 88(10), 1549–1563. 10.1111/1365-2656.13052 31310340

[gcb16140-bib-0063] Gibb, H. , Sanders, N. J. , Dunn, R. R. , Arnan, X. , Vasconcelos, H. L. , Donoso, D. A. , Andersen, A. N. , Silva, R. R. , Bishop, T. R. , Gomez, C. , Grossman, B. F. , Yusah, K. M. , Luke, S. H. , Pacheco, R. , Pearce‐Duvet, J. , Retana, J. , Tista, M. , & Parr, C. L. (2017). Habitat disturbance selects against both small and large species across varying climates. Ecography, 41(7), 1184–1193. 10.1111/ecog.03244

[gcb16140-bib-0064] Gibb, H. , Sanders, N. J. , Dunn, R. R. , Watson, S. , Photakis, M. , Abril, S. , Andersen, A. N. , Angulo, E. , Armbrecht, I. , Arnan, X. , Baccaro, F. B. , Bishop, T. R. , Boulay, R. , Castracani, C. , Del Toro, I. , Delsinne, T. , Diaz, M. , Donoso, D. A. , Enríquez, M. L. , … Parr, C. L. (2015). Climate mediates the effects of disturbance on ant assemblage structure. Proceedings of the Royal Society of London B: Biological Sciences, 282(1808), 20150418. 10.1098/rspb.2015.0418 PMC4455809 25994675

[gcb16140-bib-0065] Gras, P. , Tscharntke, T. , Maas, B. , Tjoa, A. , Hafsah, A. , & Clough, Y. (2016). How ants, birds and bats affect crop yield along shade gradients in tropical cacao agroforestry. Journal of Applied Ecology, 53(3), 953–963. 10.1111/1365-2664.12625

[gcb16140-bib-0066] Greenaway, P. (1981). Temperature limits to trailing activity in the Australian arid‐zone meat ant Iridomyrmex pupureus form viridiaeneus. Australian Journal of Zoology, 29(4), 621–630. 10.1071/ZO9810621

[gcb16140-bib-0067] Griffiths, H. M. , Ashton, L. A. , Walker, A. E. , Hasan, F. , Evans, T. A. , Eggleton, P. , & Parr, C. L. (2018). Ants are the major agents of resource removal from tropical rainforests. Journal of Animal Ecology, 87, 293–300. 10.1111/1365-2656.12728 28791685 PMC6849798

[gcb16140-bib-0068] Guo, F. , Guénard, B. , Economo, E. P. , Deutsch, C. A. , & Bonebrake, T. C. (2020). Activity niches outperform thermal physiological limits in predicting global ant distributions. Journal of Biogeography, 47(4), 829–842. 10.1111/jbi.13799

[gcb16140-bib-0069] Heller, N. E. , & Gordon, D. M. (2006). Seasonal spatial dynamics and causes of nest movement in colonies of the invasive Argentine ant (Linepithema humile). Ecological Entomology, 31(5), 499–510. 10.1111/j.1365-2311.2006.00806.x

[gcb16140-bib-0070] Helms, J. A. (2018). The flight ecology of ants (Hymenoptera: Formicidae). Myrmecological News, 26, 19–30.

[gcb16140-bib-0071] Hölldobler, B. , & Wilson, E. O. (1990). The Ants. Springer‐Verlag.

[gcb16140-bib-0072] Hölldobler, B. , & Wilson, E. O. (2009). The superorganism: The beauty, elegance, and strangeness of insect societies. W.W. Norton.

[gcb16140-bib-0073] Hood, W. G. , & Tschinkel, W. R. (1990). Desiccation resistance in arboreal and terrestrial ants. Physiological Entomology, 15(1), 23–35. 10.1111/j.1365-3032.1990.tb00489.x

[gcb16140-bib-0074] Houadria, M. , & Menzel, F. (2020). Temporal and dietary niche is context‐dependent in tropical ants. Ecological Entomology, 45(4), 761–770. 10.1111/een.12857

[gcb16140-bib-0075] Hurlbert, A. H. , Ballantyne, F. , & Powell, S. (2008). Shaking a leg and hot to trot: the effects of body size and temperature on running speed in ants. Ecological Entomology, 33(1), 144–154. 10.1111/j.1365-2311.2007.00962.x

[gcb16140-bib-0076] Jayatilaka, P. , Narendra, A. , Reid, S. F. , Cooper, P. , & Zeil, J. (2011). Different effects of temperature on foraging activity schedules in sympatric Myrmecia ants. The Journal of Experimental Biology, 214(16), 2730–2738. 21795570 10.1242/jeb.053710

[gcb16140-bib-0077] Jenkins, C. N. , Sanders, N. J. , Andersen, A. N. , Arnan, X. , Brühl, C. A. , Cerda, X. , Ellison, A. M. , Fisher, B. L. , Fitzpatrick, M. C. , Gotelli, N. J. , Gove, A. D. , Guénard, B. , Lattke, J. E. , Lessard, J.‐P. , McGlynn, T. P. , Menke, S. B. , Parr, C. L. , Philpott, S. M. , Vasconcelos, H. L. , … Dunn, R. R. (2011). Global diversity in light of climate change: the case of ants. Diversity and Distributions, 17(4), 652–662. 10.1111/j.1472-4642.2011.00770.x

[gcb16140-bib-0078] Jones, J. C. , & Oldroyd, B. P. (2006). Nest thermoregulation in social insects. Advances in Insect Physiology, 33, 153–191.

[gcb16140-bib-0079] Jordano, D. , & Thomas, C. (1992). Specificity of an ant‐lycaenid interaction. Oecologia, 91(3), 431–438. 10.1007/BF00317634 28313553

[gcb16140-bib-0080] Joseph, G. S. , Muluvhahothe, M. M. , Seymour, C. L. , Munyai, T. C. , Bishop, T. R. , & Foord, S. H. (2019). Stability of Afromontane ant diversity decreases across an elevation gradient. Global Ecology and Conservation, 17, e00596. 10.1016/j.gecco.2019.e00596

[gcb16140-bib-0081] Kadochová, Š. , & Frouz, J. (2013). Thermoregulation strategies in ants in comparison to other social insects, with a focus on red wood ants (Formica Rufa group). F1000Research, 2. 10.12688/f1000research.2-280.v1 PMC3962001 24715967

[gcb16140-bib-0082] Kaplan, I. , & Eubanks, M. D. (2005). Aphids alter the community‐wide impact of fire ants. Ecology, 86(6), 1640–1649. 10.1890/04-0016

[gcb16140-bib-0083] Kaspari, M. , Bujan, J. , Roeder, K. A. , de Beurs, K. , & Weiser, M. D. (2019). Species energy and thermal performance theory predict 20‐yr changes in ant community abundance and richness. Ecology, 100, e02888. 10.1002/ecy.2888 31505036

[gcb16140-bib-0084] Kaspari, M. , Clay, N. A. , Lucas, J. , Yanoviak, S. P. , & Kay, A. (2015). Thermal adaptation generates a diversity of thermal limits in a rainforest ant community. Global Change Biology, 21(3), 1092–1102. 10.1111/gcb.12750 25242246

[gcb16140-bib-0085] Kleineidam, C. , & Roces, F. (2000). Carbon dioxide concentrations and nest ventilation in nests of the leaf‐cutting ant Atta vollenweideri. Insectes Sociaux, 47(3), 241–248. 10.1007/PL00001710

[gcb16140-bib-0086] Kremer, J. M. , Nooten, S. S. , Cook, J. M. , Ryalls, J. M. , Barton, C. V. , & Johnson, S. N. (2018). Elevated atmospheric carbon dioxide concentrations promote ant tending of aphids. Journal of Animal Ecology, 87(5), 1475–1483. 10.1111/1365-2656.12842 29700820

[gcb16140-bib-0087] Law, S. J. , Bishop, T. R. , Eggleton, P. , Griffiths, H. , Ashton, L. , & Parr, C. L. (2020). Darker ants dominate the canopy: Testing macroecological hypotheses for patterns in colour along a microclimatic gradient. Journal of Animal Ecology, 89(2), 347–359. 10.1111/1365-2656.13110 31637702 PMC7027836

[gcb16140-bib-0088] Lucky, A. , Trautwein, M. D. , Guénard, B. S. , Weiser, M. D. , & Dunn, R. R. (2013). Tracing the rise of ants—Out of the ground. PLoS One, 8(12), e84012. 10.1371/journal.pone.0084012 24386323 PMC3873401

[gcb16140-bib-0089] MacMahon, J. A. , Mull, J. F. , & Crist, T. O. (2000). Harvester ants (Pogonomyrmex spp.): Their community and ecosystem influences. Annual Review of Ecology and Systematics, 31(1), 265–291.

[gcb16140-bib-0090] Martin, R. A. , Chick, L. D. , Garvin, M. L. , & Diamond, S. E. (2021). In a nutshell, a reciprocal transplant experiment reveals local adaptation and fitness trade‐offs in response to urban evolution in an acorn‐dwelling ant. Evolution, 75(4), 876–887. 10.1111/evo.14191 33586171 PMC8247984

[gcb16140-bib-0091] Martin, R. A. , Chick, L. D. , Yilmaz, A. R. , & Diamond, S. E. (2019). Evolution, not transgenerational plasticity, explains the adaptive divergence of acorn ant thermal tolerance across an urban–rural temperature cline. Evolutionary Applications, 12(8), 1678–1687. 10.1111/eva.12826 31462922 PMC6708418

[gcb16140-bib-0092] McGlynn, T. P. (2012). The ecology of nest movement in social insects. Annual Review of Entomology, 57, 291–308. 10.1146/annurev-ento-120710-100708 21910641

[gcb16140-bib-0093] McGlynn, T. P. , Dunn, T. , Wayman, E. , & Romero, A. (2010). A thermophile in the shade: Light‐directed nest relocation in the Costa Rican ant Ectatomma ruidum. Journal of Tropical Ecology, 26(5), 559–562.

[gcb16140-bib-0094] McMunn, M. , & Pepi, A. (2022). Predicted asymmetrical effects of warming on nocturnal and diurnal soil‐dwelling ectotherms. The American Naturalist, 199(2), 302–312. 10.1086/717431 35077281

[gcb16140-bib-0095] Menke, S. B. , Harte, J. , & Dunn, R. R. (2014). Changes in ant community composition caused by 20 years of experimental warming vs. 13 years of natural climate shift. Ecosphere, 5(1), 1–17.

[gcb16140-bib-0096] Merilä, J. , & Hendry, A. P. (2014). Climate change, adaptation, and phenotypic plasticity: The problem and the evidence. Evolutionary Applications, 7(1), 1–14. 10.1111/eva.12137 24454544 PMC3894893

[gcb16140-bib-0097] Mooney, E. , Davidson, B. , Den Uyl, J. , Mullins, M. , Medina, E. , Nguyen, P. , & Owens, J. (2019). Elevated temperatures alter an ant‐aphid mutualism. Entomologia Experimentalis Et Applicata, 167(10), 891–905. 10.1111/eea.12839

[gcb16140-bib-0098] Moreau, C. S. , Bell, C. D. , Vila, R. , Archibald, S. B. , & Pierce, N. E. (2006). Phylogeny of the ants: Diversification in the age of angiosperms. Science, 312(5770), 101–104. 10.1126/science.1124891 16601190

[gcb16140-bib-0099] Ness, J. , Mooney, K. , & Lach, L. (2010). Ants as mutualists. In L. Lach , C. L. Parr , & K. L. Abbott (Eds.), Ant ecology (pp. 97–114). Oxford University Press.

[gcb16140-bib-0100] Nkem, J. N. , Lobry de Bruyn, L. A. , Grant, C. D. , & Hulugalle, N. R. (2000). The impact of ant bioturbation and foraging activities on surrounding soil properties. Pedobiologia, 44(5), 609–621. 10.1078/S0031-4056(04)70075-X

[gcb16140-bib-0101] O'Dowd, D. J. , Green, P. T. , & Lake, P. S. (2003). Invasional ‘meltdown’on an oceanic island. Ecology Letters, 6(9), 812–817. 10.1046/j.1461-0248.2003.00512.x

[gcb16140-bib-0102] Oliveira, F. M. , Andersen, A. N. , Arnan, X. , Ribeiro‐Neto, J. D. , Arcoverde, G. B. , & Leal, I. R. (2019). Effects of increasing aridity and chronic anthropogenic disturbance on seed dispersal by ants in Brazilian Caatinga. Journal of Animal Ecology, 88(6), 870–880. 10.1111/1365-2656.12979 30883729

[gcb16140-bib-0103] Olson, D. M. , Dinerstein, E. , Wikramanayake, E. D. , Burgess, N. D. , Powell, G. V. N. , Underwood, E. C. , D'amico, J. A. , Itoua, I. , Strand, H. E. , Morrison, J. C. , Loucks, C. J. , Allnutt, T. F. , Ricketts, T. H. , Kura, Y. , Lamoreux, J. F. , Wettengel, W. W. , Hedao, P. , & Kassem, K. R. (2001). Terrestrial ecoregions of the world: A new map of life on eartha new global map of terrestrial ecoregions provides an innovative tool for conserving biodiversity. BioScience, 51(11), 933–938.

[gcb16140-bib-0104] Paolucci, L. N. , Schoereder, J. H. , Brando, P. M. , & Andersen, A. N. (2017). Fire‐induced forest transition to derived savannas: Cascading effects on ant communities. Biological Conservation, 214, 295–302. 10.1016/j.biocon.2017.08.020

[gcb16140-bib-0105] Parker, J. , & Kronauer, D. J. (2021). How ants shape biodiversity. Current Biology, 31(19), R1208–R1214. 10.1016/j.cub.2021.08.015 34637733

[gcb16140-bib-0106] Parr, C. L. (2008). Dominant ants can control assemblage species richness in a South African savanna. Journal of Animal Ecology, 77(6), 1191–1198. 10.1111/j.1365-2656.2008.01450.x 18637854

[gcb16140-bib-0107] Parr, C. L. , Eggleton, P. , Davies, A. , Evans, T. , & Holdsworth, S. (2016). Suppression of savanna ants alters invertebrate composition and influences key ecosystem processes. Ecology, 97(6), 1611–1617. 10.1890/15-1713.1 27459790

[gcb16140-bib-0108] Parr, C. L. , Gray, E. F. , & Bond, W. J. (2012). Cascading biodiversity and functional consequences of a global change–induced biome switch. Diversity and Distributions, 18(5), 493–503. 10.1111/j.1472-4642.2012.00882.x

[gcb16140-bib-0109] Pecl, G. T. , Araújo, M. B. , Bell, J. D. , Blanchard, J. , Bonebrake, T. C. , Chen, I.‐C. , Clark, T. D. , Colwell, R. K. , Danielsen, F. , Evengård, B. , Falconi, L. , Ferrier, S. , Frusher, S. , Garcia, R. A. , Griffis, R. B. , Hobday, A. J. , Janion‐Scheepers, C. , Jarzyna, M. A. , Jennings, S. , … Williams, S. E. (2017). Biodiversity redistribution under climate change: Impacts on ecosystems and human well‐being. Science, 355(6332). 10.1126/science.aai9214 28360268

[gcb16140-bib-0110] Pedersen, J. S. , & Boomsma, J. J. (1999). Genetic analysis of colony structure in polydomous and polygynous ant populations. Biological Journal of the Linnean Society, 66(1), 115–144. 10.1111/j.1095-8312.1999.tb01919.x

[gcb16140-bib-0111] Peeters, C. (2012). Convergent evolution of wingless reproductives across all subfamilies of ants, and sporadic loss of winged queens (Hymenoptera: Formicidae). Myrmecological News, 16, 75–91.

[gcb16140-bib-0112] Peeters, C. , & Ito, F. (2015). Wingless and dwarf workers underlie the ecological success of ants (Hymenoptera: Formicidae). Myrmecological News, 21, 117–130.

[gcb16140-bib-0113] Peeters, C. , & Molet, M. (2010). Colonial reproduction and life histories. In L. Lach , C. L. Parr , & K. L. Abbott (Eds.), Ant ecology (pp. 159–176). Oxford University Press.

[gcb16140-bib-0114] Pelini, S. L. , Bowles, F. P. , Ellison, A. M. , Gotelli, N. J. , Sanders, N. J. , & Dunn, R. R. (2011). Heating up the forest: open‐top chamber warming manipulation of arthropod communities at Harvard and Duke Forests. Methods in Ecology and Evolution, 2(5), 534–540. 10.1111/j.2041-210X.2011.00100.x

[gcb16140-bib-0115] Pelini, S. L. , Diamond, S. E. , MacLean, H. , Ellison, A. M. , Gotelli, N. J. , Sanders, N. J. , & Dunn, R. R. (2012). Common garden experiments reveal uncommon responses across temperatures, locations, and species of ants. Ecology and Evolution, 2(12), 3009–3015. 10.1002/ece3.407 23301168 PMC3538996

[gcb16140-bib-0116] Pelini, S. L. , Diamond, S. E. , Nichols, L. M. , Stuble, K. L. , Ellison, A. M. , Sanders, N. J. , Dunn, R. R. , & Gotelli, N. J. (2014). Geographic differences in effects of experimental warming on ant species diversity and community composition. Ecosphere, 5(10), 1–12. 10.1890/ES14-00143.1

[gcb16140-bib-0117] Penick, C. A. , Diamond, S. E. , Sanders, N. J. , & Dunn, R. R. (2017). Beyond thermal limits: comprehensive metrics of performance identify key axes of thermal adaptation in ants. Functional Ecology, 31(5), 1091–1100. 10.1111/1365-2435.12818

[gcb16140-bib-0118] Penick, C. A. , Savage, A. M. , & Dunn, R. R. (2015). Stable isotopes reveal links between human food inputs and urban ant diets. Proceedings of the Royal Society B: Biological Sciences, 282(1806), 20142608. 10.1098/rspb.2014.2608 PMC4426608 25833850

[gcb16140-bib-0119] Penick, C. A. , & Tschinkel, W. (2008). Thermoregulatory brood transport in the fire ant, Solenopsis Invicta. Insectes Sociaux, 55(2), 176–182. 10.1007/s00040-008-0987-4

[gcb16140-bib-0120] Pfeiffer, M. , Mezger, D. , & Dyckmans, J. (2014). Trophic ecology of tropical leaf litter ants (Hymenoptera: Formicidae)–a stable isotope study in four types of Bornean rain forest. Myrmecological News, 19, 31–41.

[gcb16140-bib-0121] Piñol, J. , Espadaler, X. , Cañellas, N. , Martínez‐Vilalta, J. , Barrientos, J. A. , & Sol, D. (2010). Ant versus bird exclusion effects on the arthropod assemblage of an organic citrus grove. Ecological Entomology, 35(3), 367–376. 10.1111/j.1365-2311.2010.01190.x

[gcb16140-bib-0122] Pontin, A. (1960). Field experiments on colony foundation by Lasius niger (L.) and L. flavus (F.)(Hym., Formicidae). Insectes Sociaux, 7(3), 227–230. 10.1007/BF02224494

[gcb16140-bib-0123] Porter, S. D. (1988). Impact of temperature on colony growth and developmental rates of the ant, Solenopsis invicta. Journal of Insect Physiology, 34(12), 1127–1133. 10.1016/0022-1910(88)90215-6

[gcb16140-bib-0124] Pringle, E. G. , Akçay, E. , Raab, T. K. , Dirzo, R. , & Gordon, D. M. (2013). Water stress strengthens mutualism among ants, trees, and scale insects. PLoS Biology, 11(11), e1001705. 10.1371/journal.pbio.1001705 24223521 PMC3818173

[gcb16140-bib-0125] Reichman, O. , & Seabloom, E. W. (2002). The role of pocket gophers as subterranean ecosystem engineers. Trends in Ecology & Evolution, 17(1), 44–49. 10.1016/S0169-5347(01)02329-1

[gcb16140-bib-0126] Rico‐Gray, V. , & Oliveira, P. S. (2007). The ecology and evolution of ant‐plant interactions. University of Chicago Press.

[gcb16140-bib-0127] Roces, F. , & Núñez, J. A. (1989). Brood translocation and circadian variation of temperature preference in the ant Camponotus mus. Oecologia, 81(1), 33–37. 10.1007/BF00377006 28312153

[gcb16140-bib-0128] Roeder, D. V. , Paraskevopoulos, A. W. , & Roeder, K. A. (2022). Thermal tolerance regulates foraging behaviour of ants. Ecological Entomology. 10.1111/een.13118

[gcb16140-bib-0129] Roeder, K. A. , Bujan, J. , de Beurs, K. M. , Weiser, M. D. , & Kaspari, M. (2021a). Thermal traits predict the winners and losers under climate change: an example from North American ant communities. Ecosphere, 12(7), e03645. 10.1002/ecs2.3645

[gcb16140-bib-0130] Roeder, K. A. , Roeder, D. V. , & Bujan, J. (2021b). Ant Thermal tolerance: A review of methods, hypotheses, and sources of variation. Annals of the Entomological Society of America, 114(4), 459–469. 10.1093/aesa/saab018

[gcb16140-bib-0131] Römer, D. , Bollazzi, M. , & Roces, F. (2017). Carbon dioxide sensing in an obligate insect‐fungus symbiosis: CO_2_ preferences of leaf‐cutting ants to rear their mutualistic fungus. PLoS One, 12(4), e0174597. 10.1371/journal.pone.0174597 28376107 PMC5380341

[gcb16140-bib-0132] Römer, D. , Bollazzi, M. , & Roces, F. (2018). Carbon dioxide sensing in the social context: Leaf‐cutting ants prefer elevated CO2 levels to tend their brood. Journal of Insect Physiology, 108, 40–47. 10.1016/j.jinsphys.2018.05.007 29778905

[gcb16140-bib-0133] Sanada‐Morimura, S. , Satoh, T. , & Obara, Y. (2006). Territorial behavior and temperature preference for nesting sites in a pavement ant Tetramorium tsushimae. Insectes Sociaux, 53(2), 141–148. 10.1007/s00040-005-0849-2

[gcb16140-bib-0134] Sanders, D. , & van Veen, F. J. F. (2011). Ecosystem engineering and predation: The multi‐trophic impact of two ant species. Journal of Animal Ecology, 80(3), 569–576. 10.1111/j.1365-2656.2010.01796.x 21244419

[gcb16140-bib-0135] Sanders, N. J. , Lessard, J.‐P. , Fitzpatrick, M. C. , & Dunn, R. R. (2007). Temperature, but not productivity or geometry, predicts elevational diversity gradients in ants across spatial grains. Global Ecology and Biogeography, 16(5), 640–649. 10.1111/j.1466-8238.2007.00316.x

[gcb16140-bib-0136] Schmidt, C. A. , & Shattuck, S. O. (2014). The higher classification of the ant subfamily Ponerinae (Hymenoptera: Formicidae), with a review of ponerine ecology and behavior. Zootaxa, 3817(1), 1–242. 24943802 10.11646/zootaxa.3817.1.1

[gcb16140-bib-0137] Shi, N. N. , Tsai, C.‐C. , Camino, F. , Bernard, G. D. , Yu, N. , & Wehner, R. (2015). Keeping cool: Enhanced optical reflection and heat dissipation in silver ants. Science, 349, 298–301. 26089358 10.1126/science.aab3564

[gcb16140-bib-0138] Shik, J. Z. , Arnan, X. , Oms, C. S. , Cerdá, X. , & Boulay, R. (2019). Evidence for locally adaptive metabolic rates among ant populations along an elevational gradient. Journal of Animal Ecology, 88(8), 1240–1249. 10.1111/1365-2656.13007 31077366

[gcb16140-bib-0139] Sinclair, B. J. , Ferguson, L. V. , Salehipour‐Shirazi, G. , & MacMillan, H. A. (2013). Cross‐tolerance and cross‐talk in the cold: relating low temperatures to desiccation and immune stress in insects. Integrative and Comparative Biology, 53(4), 545–556. 10.1093/icb/ict004 23520401

[gcb16140-bib-0140] Soare, T. W. , Kumar, A. , Naish, K. A. , & O'Donnell, S. (2014). Genetic evidence for landscape effects on dispersal in the army ant Eciton burchellii. Molecular Ecology, 23(1), 96–109. 24372755 10.1111/mec.12573

[gcb16140-bib-0141] Sommer, S. , & Wehner, R. (2012). Leg allometry in ants: Extreme long‐leggedness in thermophilic species. Arthropod Structure & Development, 41(1), 71–77. 10.1016/j.asd.2011.08.002 21992805

[gcb16140-bib-0142] Stuble, K. L. , Juric, I. , Cerda, X. , & Sanders, N. J. (2017). Dominance hierarchies are a dominant paradigm in ant ecology (Hymenoptera: Formicidae), but should they be? and what is a dominance hierarchy anyways. Myrmecological News, 24, 71–81.

[gcb16140-bib-0143] Stuble, K. L. , Patterson, C. M. , Rodriguez‐Cabal, M. A. , Ribbons, R. R. , Dunn, R. R. , & Sanders, N. J. (2014). Ant‐mediated seed dispersal in a warmed world. PeerJ, 2, e286. 10.7717/peerj.286 24688863 PMC3961163

[gcb16140-bib-0144] Stuble, K. L. , Pelini, S. L. , Diamond, S. E. , Fowler, D. A. , Dunn, R. R. , & Sanders, N. J. (2013). Foraging by forest ants under experimental climatic warming: A test at two sites. Ecology and Evolution, 3(3), 482–491. 10.1002/ece3.473 23531642 PMC3605839

[gcb16140-bib-0145] Styrsky, J. D. , & Eubanks, M. D. (2007). Ecological consequences of interactions between ants and honeydew‐producing insects. Proceedings of the Royal Society B: Biological Sciences, 274(1607), 151–164. 10.1098/rspb.2006.3701 PMC1685857 17148245

[gcb16140-bib-0146] Szewczyk, T. , & McCain, C. M. (2016). A Systematic Review of global drivers of ant elevational diversity. PLoS One, 11(5), e0155404. 10.1371/journal.pone.0155404 27175999 PMC4866765

[gcb16140-bib-0147] Tamashiro, R. A. , Milligan, P. D. , & Palmer, T. M. (2019). Left out in the cold: temperature‐dependence of defense in an African ant–plant mutualism. Ecology, 100, e02712. 10.1002/ecy.2712 31095732

[gcb16140-bib-0148] Tocco, C. , Foster, J. , Venter, N. , Cowie, B. , Marlin, D. , & Byrne, M. (2021). Elevated atmospheric CO_2_ adversely affects a dung beetle’s development: Another potential driver of decline in insect numbers? Global Change Biology. 10.1111/gcb.15804 34265139

[gcb16140-bib-0149] Tschinkel, W. R. (1987). Seasonal life history and nest architecture of a winter‐active ant, Prenolepis Imparis. Insectes Sociaux, 34(3), 143–164. 10.1007/BF02224081

[gcb16140-bib-0150] Tuma, J. , Eggleton, P. , & Fayle, T. M. (2020). Ant‐termite interactions: An important but under‐explored ecological linkage. Biological Reviews, 95, 555–572. 10.1111/brv.12577 31876057

[gcb16140-bib-0151] Vanderplank, F. (1960). The bionomics and ecology of the red tree ant, Oecophylla sp., and its relationship to the coconut bug Pseudotheraptus wayi Brown (Coreidae). Journal of Animal Ecology, 29, 15–33. 10.2307/2268

[gcb16140-bib-0152] Verberk, W. C. , Atkinson, D. , Hoefnagel, K. N. , Hirst, A. G. , Horne, C. R. , & Siepel, H. (2021). Shrinking body sizes in response to warming: Explanations for the temperature–size rule with special emphasis on the role of oxygen. Biological Reviews, 96(1), 247–268. 10.1111/brv.12653 32959989 PMC7821163

[gcb16140-bib-0153] Villalta, I. , Oms, C. S. , Angulo, E. , Molinas‐González, C. R. , Devers, S. , Cerdá, X. , & Boulay, R. (2020). Does social thermal regulation constrain individual thermal tolerance in an ant species? Journal of Animal Ecology, 89(9), 2063–2076. 10.1111/1365-2656.13268 32445419

[gcb16140-bib-0154] Vogt, J. T. , Streett, D. A. , & Boykin, D. (2004). Seasonal characteristics of black imported fire ant (Hymenoptera: Formicidae) mounds in northern Mississippi pastures. Sociobiology, 43, 513–522.

[gcb16140-bib-0155] Warren, R. J. , & Bradford, M. A. (2014). Mutualism fails when climate response differs between interacting species. Global Change Biology, 20(2), 466–474. 10.1111/gcb.12407 24399754

[gcb16140-bib-0156] Warren, R. , Chick, L. , DeMarco, B. , McMillan, A. , De Stefano, V. , Gibson, R. , & Pinzone, P. (2016). Climate‐driven range shift prompts species replacement. Insectes Sociaux, 63(4), 593–601. 10.1007/s00040-016-0504-0

[gcb16140-bib-0157] Warren, R. J. , Mathew, A. , Reed, K. , Bayba, S. , Krupp, K. , & Spiering, D. J. (2019). Myrmica rubra microhabitat selection and putative ecological impact. Ecological Entomology, 44(2), 239–248.

[gcb16140-bib-0158] Wehner, R. , & Wehner, S. (2011). Parallel evolution of thermophilia: daily and seasonal foraging patterns of heat‐adapted desert ants: Cataglyphis and Ocymyrmex species. Physiological Entomology, 36(3), 271–281. 10.1111/j.1365-3032.2011.00795.x

[gcb16140-bib-0159] Wilkinson, M. T. , Richards, P. J. , & Humphreys, G. S. (2009). Breaking ground: pedological, geological, and ecological implications of soil bioturbation. Earth‐Science Reviews, 97(1–4), 257–272. 10.1016/j.earscirev.2009.09.005

[gcb16140-bib-0160] Williamson, C. E. , Zepp, R. G. , Lucas, R. M. , Madronich, S. , Austin, A. T. , Ballaré, C. L. , Norval, M. , Sulzberger, B. , Bais, A. F. , McKenzie, R. L. , Robinson, S. A. , Häder, D.‐P. , Paul, N. D. , & Bornman, J. F. (2014). Solar ultraviolet radiation in a changing climate. Nature Climate Change, 4(6), 434–441. 10.1038/nclimate2225

[gcb16140-bib-0161] Willmer, P. , & Unwin, D. (1981). Field analyses of insect heat budgets: Reflectance, size and heating rates. Oecologia, 50(2), 250–255. 10.1007/BF00348047 28311097

[gcb16140-bib-0162] Wilson, E. O. (1990). Success and dominance in ecosystems: The case of the social insects. Ecology Institute.

[gcb16140-bib-0163] Wilson, E. O. , & Hölldobler, B. (2005). The rise of the ants: A phylogenetic and ecological explanation. Proceedings of the National Academy of Sciences of the United States of America, 102(21), 7411–7414. 10.1073/pnas.0502264102 15899976 PMC1140440

[gcb16140-bib-0164] Youngsteadt, E. , Henderson, R. C. , Savage, A. M. , Ernst, A. F. , Dunn, R. R. , & Frank, S. D. (2015). Habitat and species identity, not diversity, predict the extent of refuse consumption by urban arthropods. Global Change Biology, 21(3), 1103–1115. 10.1111/gcb.12791 25463151

[gcb16140-bib-0165] Zhou, A. , Qu, X. , Shan, L. , & Wang, X. (2017). Temperature warming strengthens the mutualism between ghost ants and invasive mealybugs. Scientific Reports, 7(1), 1–10. 10.1038/s41598-017-01137-0 28424508 PMC5430489

